# Structural validity of the German WISC-V beyond primary assessment: a factor-analytical approach to the ancillary index scales

**DOI:** 10.3389/fpsyg.2026.1913590

**Published:** 2026-09-03

**Authors:** Franz Pauls, Monika Daseking

**Affiliations:** 1 Clinical Psychology and Psychotherapy, Helmut-Schmidt-University/University of the Federal Armed Forces, Hamburg, Germany; 2 Department of Developmental and Educational Psychology, Helmut-Schmidt-University/University of the Federal Armed Forces, Hamburg, Germany

**Keywords:** ancillary index scales, confirmatory factor analysis, extended German standardization sample, intelligence assessment, structural validity, WISC-V

## Abstract

**Introduction:**

Aligning with the factor-analytical procedure already established for the original test version, the present study examined the structural validity and diagnostic viability of the German *Wechsler Intelligence Scale for Children—Fifth Edition* (WISC-V) ancillary index scales on an extended standardization sample of children and adolescents aged between 6 and 16 years (*N* = 1,466).

**Methods:**

Representing the first comprehensive investigation into the ancillary index structure of the German WISC-V, competing hierarchical and bifactor model solutions were systematically evaluated via confirmatory factor analyses (CFA) and supplemented by model-based reliability analyses.

**Results:**

The results provided structural support for the retention of the *Auditory Working Memory* index (AWM) as a structurally identifiable yet psychometrically redundant scale, whereas a subordinate factor for the *Quantitative Reasoning* index (QR) was completely unviable due to estimation failures and non-convergence across both model frameworks. A hierarchical model solution confirmed that the *Nonverbal Index* (NVI) represents a valid multidimensional construct, though model-based reliability analyses indicated that its underlying specific subscales lack the structural stability for independent interpretation beyond the NVI composite score. Furthermore, model-fit evaluations established that the *General Ability Index* (GAI) and the *Cognitive Proficiency Index* (CPI) are best represented by a second-order oblique four-factor model solution. While the GAI and CPI are reflected by plausible latent dimensions, their profound statistical proximity to the *Full Scale Intelligence Quotient* (FSIQ) and the underlying subscales’ lack of sufficient statistical stability underscore the risk of profile misinterpretation.

**Discussion:**

Consequently, study limitations and boundaries for evidence-based assessment are discussed, emphasizing that subsequent diagnostic decision-making in clinical practice based on the ancillary index scales alone should be approached with caution absent further cross-cultural and clinical validation.

## 1 Introduction

As a cornerstone of contemporary intelligence assessment, the *Wechsler Intelligence Scale for Children–Fifth Edition* (WISC-V; Wechsler, 2014a, 2017a) continues to be a widely utilized instrument for evaluating cognitive abilities in children and adolescents aged between 6 and 16 years. Grounded in the Cattell-Horn-Carroll (CHC) model of intelligence (McGrew, 2009; Schneider and McGrew, 2012), the WISC-V represents a significant evolution from its predecessor, as the test battery redefines the four-factor model of the WISC-IV (Wechsler, 2003, 2009) as a new primary five-factor measurement model by splitting the former factor *Perceptual Reasoning* (PR) into two distinct structural domains: *Visual Spatial* (VS) and *Fluid Reasoning* (FR). This key modification results in a revised scoring framework, which describes the *Full Scale Intelligence Quotient* (FSIQ) as an estimate of general intelligence based on five cognitive subdomain indices, each contributing in varying proportions to the total score: *Verbal Comprehension Index* (VCI), *Visual Spatial Index* (VSI), *Fluid Reasoning Index* (FRI), *Working Memory Index* (WMI), and *Processing Speed Index* (PSI). Each of the corresponding five primary index scores is derived using two out of ten primary subtests: Vocabulary (VO), Similarities (SI), Block Design (BD), Visual Puzzles (VP), Matrix Reasoning (MR), Figure Weights (FW), Digit Span (DS), Picture Span (PS), Coding (CD), and Symbol Search (SS). Beyond these primary subtests, the WISC-V additionally includes six secondary subtests: Information (IN), Comprehension (CO), Picture Concepts (PC), Arithmetic (AR), Letter-Number Sequencing (LN), and Cancellation (CA). These secondary subtests can be further integrated with specific primary subtests to obtain five ancillary index scores for the *General Ability Index* (GAI), *Nonverbal Index* (NVI), *Cognitive Proficiency Index* (CPI), *Auditory Working Memory* (AWM), and *Quantitative Reasoning* (QR). The organizational and scoring framework of the WISC-V is outlined in Supplementary Table S1.

### 1.1 Structural framework of the WISC-V

Preliminary structural validity of the WISC-V was established by the test publisher through a series of confirmatory factor analyses (CFA) comparing alternative factor model solutions to identify a theoretically sound primary measurement model for the standardization data. Their analyses indicated that hierarchical five-factor model solutions were generally superior to four-factor model alternatives. Consequently, a second-order five-factor model was selected to represent the WISC-V structure, featuring a hierarchical (second-order) general intelligence factor (*g*) influencing subtests indirectly through five first-order factors (see Wechsler, 2014b, for a detailed description). However, since the initial publication of the WISC-V, independent scholars have increasingly questioned the veracity of the structural and interpretive model proposed by the test publisher (e.g., Beaujean, 2016; Canivez and Watkins, 2016).

Besides failures in fully disclosing important details about the CFA model specifications used, one of several concerns was that the test publisher relied exclusively on CFA to evaluate internal structure, thus neglecting results provided by complementary exploratory factor analyses (EFA). Furthermore, these procedures produced a final factor model containing many of the same problematic parameters associated with prior attempts to explicate a five-factor model structure for the WISC-IV (e.g., Weiss et al., 2013). While some research has provided support for a five-factor model structure both in normative (e.g., Keith et al., 2006) and clinical samples (e.g., Chen et al., 2015; Reynolds and Keith, 2017), an ongoing debate persists regarding whether subtest performances are more accurately described by a four- or five-factor model framework (Dombrowski et al., 2018). Specifically, independent CFA and EFA studies have frequently indicated that a rival four-factor model—consistent with previous Wechsler editions—best explains the WISC-V normative data (Canivez et al., 2016, 2017; Dombrowski et al., 2018). These findings have been replicated across various international versions, including the Canadian (Watkins et al., 2017), French (Lecerf and Canivez, 2018), and Spanish WISC-V (Fenollar-Cortés and Watkins, 2018). Independent investigations of the German WISC-V have also challenged the test publisher’s preferred primary measurement model, suggesting that the primary index structure may be more accurately represented by alternative model solutions (Pauls and Daseking, 2021; Pauls et al., 2020). However, Dombrowski et al. (2021) demonstrated that while the four-factor model fitted the data best, multiple versions of a five-factor model solution were more replicable, suggesting that the four-factor model may lack stability. Another critical point of this methodological debate relates to the failure to consider rival bifactor models when comparing alternative model solutions (Beaujean, 2015). Unlike hierarchical models, where the superordinate general factor *g* is fully mediated by lower-order factors, bifactor models specify *g* and the group factors at the same level of inference, featuring independent associations with subtest indicators (Canivez and Watkins, 2016; Reise, 2012).

Although the understanding of the WISC-V primary measurement model has evolved through these independent investigations—including extensive structural analyses of the German version (Becker et al., 2024; Pauls and Daseking, 2021; Pauls et al., 2020)—the structural validity of the posited ancillary index scales has remained strikingly absent from both the *WISC-V Technical and Interpretive Manual* and subsequent independent CFA/EFA studies on the WISC-V. Since ancillary indices are intended to reflect cognitive dimensions nested within an expanded Wechsler measurement model (Weiss et al., 2016), evaluating their internal factor structure is crucial for validating their practical application. While McGill et al. (2025) recently provided a first comprehensive structural investigation of the WISC-V ancillary index scales using the US normative sample, the psychometric robustness of these scales remains a subject of interest for the German WISC-V. Therefore, a closer examination of their development and the theoretical rationale behind their specification is necessary to understand how the ancillary indices are expected to function within the overall structural framework of the German WISC-V.

### 1.2 Conceptual framework of the ancillary indices

A fundamental distinction within the conceptual framework of the WISC-V lies in how its primary and ancillary indices are derived. While the primary index scales are established through factor-analytic procedures based on the ten primary subtests in line with psychometric best practice (Keith et al., 2006), the ancillary index scales represent more “logical-formative” constructs. Thus, the ancillary indices *Quantitative Reasoning* (QR), *Auditory Working Memory* (AWM), *Nonverbal Index* (NVI), *General Ability Index* (GAI), and *Cognitive Proficiency Index* (CPI) are based on a reconfiguration of primary and secondary subtests into specific clusters to help address certain clinical questions. Specifically, QR, derived from the subtests FW and AR, and AWM, composed of the subtests DS and LN, reflect narrow abilities, while NVI provides a broad measure of ability by combining a set of six subtests requiring minimal verbal expression (BD, VP, MR, FW, PS, and CD). In contrast, the GAI and the CPI provide a bifurcated view of global cognitive functioning. While the GAI aggregates five subtests focusing on reasoning and knowledge (SI, VO, BD, MR, and FW), the CPI measures the efficiency of information processing by combining four subtests (DS, PS, CD, and SS). As noted by McGill et al. (2025), this reconfiguration of subtests into ancillary indices suggests the existence of latent dimensions that go beyond the primary measurement model of the WISC-V.

Unlike QR, AWM, and NVI, which are new to the WISC-V, the GAI and CPI have a long clinical history that predates their formal inclusion in the WISC-V. The GAI was originally introduced as a post-hoc measure for the WISC-III (Wechsler, 1991) and the WISC-IV to provide a reliable estimate of general ability in cases where other measures of general ability (i.e., the FSIQ) could be potentially confounded by influences of working memory and processing speed deficits (Prifitera et al., 1998). In clinical practice, the GAI was thus frequently utilized in the context of specific learning disability (SLD) diagnostics, aiming to prevent the FSIQ from being artificially decreased by lower performances on subtests measuring working memory capacity and processing speed (Willis and Dumont, 2002). Conversely, the CPI was later introduced to aggregate these omitted processing-dependent subtests (Dumont and Willis, 2001; Weiss et al., 2016), allowing for a direct comparison between individual reasoning ability and cognitive proficiency.

Despite their widespread clinical use, the degree to which the ancillary index scales represent distinct latent dimensions—as opposed to mere variations of *g*—is still critically debated. As highlighted by McGill et al. (2025), early attempts to integrate latent factors for the GAI and CPI as intermediary dimensions within the Wechsler structural framework (e.g., Zhu, 2009) resulted in models where both factors were virtually redundant with *g*. This redundancy suggests a high risk of model overfitting and raises doubts about whether ancillary indices in general offer incremental validity beyond the FSIQ. On top of that, the history of Wechsler-based assessment is marked by various attempts to utilize specific subtest profiles for diagnostic purposes, most of which have failed to find robust empirical support (Smith and Watkins, 2004; Styck et al., 2019). By following a rigorous factor-analytical approach to the US normative data, McGill et al. (2025) recently demonstrated that the empirical support for the structural validity of some ancillary index scales appears to be weaker than their theoretical conceptualization would suggest. Consequently, investigating whether these findings hold true for the German WISC-V is essential for clinical interpretation and decision-making processes based on the ancillary index scores.

### 1.3 Study objectives

Evaluating the interpretability of test scores based on structural validity is a fundamental requirement for the ethical and professional use of diagnostic instruments (AERA, APA, and NCME, 2014). Consequently, both researchers and clinicians should only rely on test scores of technically sound instruments once their validity has been demonstrated for the population under evaluation (Evers et al., 2013). While the factor structure of the WISC-V primary indices has been extensively scrutinized, a notable discrepancy remains between the clinical application and the psychometric evidence of its ancillary index scales. Despite their frequent application in clinical decision-making (e.g., Dale et al., 2023), these scales have remained largely unexamined in independent structural research. Furthermore, although comprehensive structural analyses of the ancillary indices have recently been conducted on the original US version (McGill et al., 2025), such in-depth investigations are still lacking for the German WISC-V. Therefore, the present study set out to investigate the latent factor structure of the WISC-V ancillary index scales by utilizing an extended, population-representative German standardization sample. Unlike previous investigations that mostly relied on summary data such as rounded correlation matrices (e.g., Canivez et al., 2021; McGill et al., 2025), the present analyses are based on original standardization data, ensuring the highest possible precision in latent factor estimation. The factor-analytical procedure of the present study is closely aligned with the approach established by McGill et al. (2025) and addresses the following three objectives:

1. First, the study examines whether the ancillary index scales AWM and QR emerge as statistically viable and distinct latent dimensions when integrated into an expanded measurement model of the German WISC-V. While McGill et al. (2025) utilized traditional higher-order models only, the present study additionally incorporates a specific bifactor modeling framework to address the first objective. Crucially, rather than assuming complete independence among all latent dimensions, this advanced extension partitions the nested, subordinate structures of AWM and QR within their primary index domains while systematically residualizing them from superordinate psychometric *g* variance. This allows for a more rigorous evaluation of whether the proposed ancillary index configurations retain sufficient specific variance to function as distinct cognitive dimensions once the dominant influence of general intelligence is controlled.
2. Regarding the structural composition of the NVI, the study investigates whether the specific set of subtests comprising this index is best reflected by a single, unidimensional latent factor. Given the differences between the US and the German WISC-V versions, it is essential to evaluate if the NVI maintains its intended structural integrity and provides a robust measure of nonverbal ability within the German cultural and linguistic context.
3. The third objective is to evaluate whether the GAI and CPI are best represented by viable intermediary dimensions within the WISC-V measurement model, extending the approach suggested by Zhu (2009). This is particularly relevant as previous evidence suggests that such composites might lack a robust reflective nature, potentially serving as redundant markers of general intelligence rather than unique cognitive domains (Edwards, 2011).

Since it is the first comprehensive investigation into the ancillary index structure of the German WISC-V, this study aims to improve the quality of data-driven decision-making in clinical and research settings by providing the empirical foundation necessary for evidence-based interpretation. While recent evidence from the US normative sample (McGill et al., 2025) has provided valuable insights into the structural validity of the WISC-V ancillary index scales, a direct cross-cultural transfer of these psychometric properties cannot be assumed *a priori*. Differences such as in linguistic adaptations, educational tracking systems, and standardization stratification variables (like those implemented in the German WISC-V) can critically alter factor loadings and subtest behavior. Furthermore, structural equation modeling analyses are often limited to evaluating global model fit. To bridge the gap between structural validity and clinical utility, a rigorous evaluation requires not only the verification of baseline and bifactor models but also the estimation of model-based reliability and replicability. Therefore, the present study extends previous research by systematically evaluating the German standardization sample using advanced confirmatory factor analysis, supplemented by model-based reliability analyses, to determine whether the ancillary scales possess sufficient unique variance to warrant independent clinical interpretation within European clinical and diagnostic guidelines.

## 2 Materials and methods

### 2.1 Participants

Data for the current analyses were derived from a newly configured subsample of the extended, population-representative German WISC-V standardization sample. A detailed description of the standardization procedures is provided in the German WISC-V *Technical and Interpretive Manual* (Wechsler, 2017b). The original standardization sample was obtained following a stratified proportional sampling protocol to mirror the recent German census for significant demographic variables, including gender, age, parental education, type of school, and migration background. This dataset was extended by additional cases, resulting in a raw dataset of 1,646 individuals (Pauls and Daseking, 2021). However, to guarantee the integrity of the latent model structures under examination, a strict case-exclusion criterion was applied: cases were only retained if complete scores necessary for the calculation of the ancillary index scales were available across all secondary subtests. Following this data-cleaning procedure, the final sample consisted of 1,466 children and adolescents (742 males; 724 females) aged between 6:0 and 16:11 years. Detailed demographic sample characteristics are summarized in Table 1. Measurement invariance across genders has already been shown for the primary measurement model of the German WISC-V using a similar dataset (Pauls et al., 2020), ensuring that the underlying subtest indicators function equivalently for males and females. To evaluate potential selection bias, the excluded subsample (*n* = 180) was systematically compared to the final sample (*N* = 1,466) using independent-samples *t*-tests and *χ*^2^-tests of independence. Results indicated no statistically significant differences between the excluded and included cases regarding the FSIQ scores [*t*(1644) = 0.95, *p* = 0.343], gender [*χ*^2^(1) = 3.29, *p* = 0.070], age [*t*(1644) = 1.36, *p* = 0.176], parental education [*χ*^2^(4) = 7.19, *p* = 0.126], or migration background [*χ*^2^(1) = 1.00, *p* = 0.317], confirming that the missingness did not introduce systematic biases.

**Table 1:** Demographic characteristics of the extended German WISC-V standardization sample (*N* = 1,466) according to gender, age, parental education, and migration background.

Gender	*N*	Age (years)			Parental education (%)					Mig. (%)
		*M*	*SD*	Min/Max	0	1	2	3	4	
Female	724	10.47	3.07	6/16	4.0	12.6	32.5	23.3	27.6	31.4
Male	742	10.44	3.06	6/16	3.9	12.3	30.9	19.9	33.0	29.2
Total	1,466	10.45	3.07	6/16	4.0	12.4	31.7	21.6	30.4	30.3

Parental education is defined as the highest level of education achieved by either one parent or both: 0 = no education, 1 = low educational level (no diploma or school certificate after 9th grade), 2 = medium educational level (school certificate after 10th grade), 3 = high educational level (school certificate after 12th or 13th grade/ university entrance qualification), 4 = highest educational level (college/university degree). Mig. (%) = percentage of cases with a migration background (migration background is indicated when either the child or at least one parent was not born in Germany).

### 2.2 Instrument and measures

The German version of the WISC-V was adapted from the original US version (WISC-V US) using a standardization kit that provides the basic framework for all European WISC-V versions. While the WISC-V US incorporates 16 subtests (Wechsler, 2014a), a major structural modification in the German version involved the complete removal of the secondary subtest Picture Concepts (PC), reducing the total available subtest pool to 15 subtests (10 primary and 5 secondary subtests) (Wechsler, 2017b). The 10 primary subtests are Vocabulary (VO), Similarities (SI), Block Design (BD), Visual Puzzles (VP), Matrix Reasoning (MR), Figure Weights (FW), Digit Span (DS), Picture Span (PS), Coding (CD), and Symbol Search (SS). While seven of these primary subtests (BD, SI, MR, DS, CD, VO, and FW) combine to form the *Full Scale Intelligence Quotient* (FSIQ), all 10 primary subtests are used to compute five primary index scores: *Verbal Comprehension Index* (VCI), *Visual Spatial Index* (VSI), *Fluid Reasoning Index* (FRI), *Working Memory Index* (WMI), and *Processing Speed Index* (PSI). To derive the five ancillary index scores for *General Ability Index* (GAI), *Cognitive Proficiency Index* (CPI), *Nonverbal Index* (NVI), *Auditory Working Memory* (AWM), and *Quantitative Reasoning* (QR), specific configurations of these primary subtests are combined with the remaining five secondary subtests: Information (IN), Comprehension (CO), Arithmetic (AR), Letter-Number Sequencing (LN), and Cancellation (CA). All 15 subtests yield scaled scores with *M* = 10 and *SD* = 3, whereas all indices and the FSIQ are defined by standard scores on the IQ scale (*M* = 100, *SD* = 15). Within the present extended German WISC-V standardization sample, all subtests showed good to excellent internal consistency, with reliability coefficients (Cronbach’s *α*) ranging from 0.80 to 0.92 for the primary subtests and from 0.82 to 0.91 for the secondary subtests.

### 2.3 Statistical analyses

Initial data screening and preliminary analyses were carried out using SPSS Statistics (Version 31.0; IBM Corp, 2025a). Model-based factor analyses (i.e., CFA) in the present study were based on the subtest covariance matrices of the extended German WISC-V standardization sample and conducted using maximum likelihood (ML) estimation within Amos (Version 31.0; IBM Corp, 2025b). Prior to model-based analyses, the subtest scaled scores were checked for normality. Univariate indices remained well within acceptable limits, adhering to the recommended thresholds of absolute skewness < 2 and absolute kurtosis < 7 (West et al., 1995). However, Mardia’s coefficient indicated significant multivariate leptokurtosis. In order to control for this biasing effect and prevent the inflation of the likelihood-ratio *χ*^2^ statistic—which can lead to the over-rejection of plausible model solutions—the Bollen-Stine bootstrapping procedure based on 2,000 resamples was implemented to obtain adjusted *p*-values (Bollen and Long, 1993; Nevitt and Hancock, 2001). To evaluate the unique substructures introduced by the inclusion of the latent factors for the ancillary index scales in the WISC-V primary measurement model, a series of competing model solutions—including both hierarchical and bifactor models—was systematically examined by conducting CFA. Bifactor models were included to systematically separate general intelligence (*g*) from group factor variance, while preserving the nested substructures within the primary ability domains. The scale of all latent variables was identified by fixing one loading of each factor to 1 (Keith, 2015). To ensure that model solutions warrant independent clinical interpretation beyond global fit indices, convergent configurations were further subjected to model-based reliability analyses.

#### 2.3.1 Model specifications

Regarding the first objective, a series of rival model solutions was specified to establish an optimal baseline measurement model for the 15 subtests of the German WISC-V. Because the theoretical classification and alignment of the Arithmetic (AR) subtest remain a subject of ongoing debate within the WISC-V structural framework (e.g., Karzmark, 2009), multiple competing CFA model configurations were systematically specified using both hierarchical and bifactor models. Prior to specifying hierarchical higher-order model configurations, standard first-order oblique models were established as psychometric baselines for both the four-factor model framework (featuring four intercorrelated first-order factors) and the five-factor model framework (featuring five intercorrelated first-order factors). Within the four-factor model framework, four hierarchical higher-order model solutions (M1a to M1d) and their four corresponding bifactor model counterparts (M1a* to M1d*) were specified. The hierarchical models M1a to M1d represent the traditional measurement models analyzed by the test publisher (Wechsler, 2017b), which cohere with different historical alignments and cross-loading hypotheses in the Wechsler interpretative literature (McGill et al., 2025). These models systematically varied the dimensional placement of AR, testing configurations where it loaded exclusively on the working memory factor or cross-loaded across multiple domains. A detailed visual representation of the structural framework for the competing hierarchical four-factor models is provided in Supplementary Table S2, while the corresponding bifactor four-factor models are illustrated in Supplementary Table S3.

To explore alternative alignments within a five-factor model framework, five hierarchical models (M2a to M2e) and a series of corresponding bifactor model counterparts (M2a* to M2e*) were examined. Like M1a to M1d, M2a to M2e represent the CFA models evaluated by the test publisher (Wechsler, 2017b). Adopting equivalent subtest-to-factor clusters, these models systematically varied the placement of AR across different latent factors. Beyond these models, two additional modified models (M2f and M2f*) were established to allow for specific structural adjustments: As proposed by Reynolds and Keith (2017), M2f was specified as a hierarchical model based on M2a but incorporated an additional correlation between FR and VS alongside a direct loading of AR on the *g*-factor, whereas M2f* represents the corresponding bifactor model counterpart featuring a correlation between the FR and VS group factors. The complete structural framework for the competing hierarchical five-factor models is visually represented in Supplementary Table S4, while the corresponding bifactor models are depicted in Supplementary Table S5. Once the optimal baseline measurement model was established from the competing CFA model solutions tested, the proposed ancillary index scales AWM and QR were subsequently integrated as minor latent factors consistent with their proposed theoretical alignment (e.g., Weiss et al., 2016) to address the first research objective.

Because the GAI, CPI, and NVI are derived from varying combinations of primary and secondary subtests, the latent factor structure for each index scale was analyzed and evaluated independently to verify its structural validity (McGill et al., 2025). For the second objective, which specifically addresses the underlying dimensionality and structural composition of the NVI, two rival model solutions were specified and evaluated. These included testing a unidimensional one-factor model (M3a) against a second-order two-factor model (M3b) to determine whether a single, undifferentiated nonverbal dimension or a structured hierarchical arrangement best captures the shared variance among the nonverbal subtest indicators. For the third objective, focusing on the structural composition and viability of the GAI and CPI, a series of five competing model frameworks was systematically evaluated: a first-order orthogonal (M4a) and an oblique (M4b) two-factor model, a second-order orthogonal (M4c) and oblique (M4d) four-factor model, and a complex third-order four-factor model (M4e) originally proposed by Zhu (2009). These models were evaluated to determine whether the GAI and CPI function as viable intermediary dimensions situated within the hierarchical model framework, or if they exhibit statistical redundancy with the superordinate *g*-factor.

In line with the modeling constraints applied by McGill et al. (2025), the consideration of additional latent factors within these higher-order CFA models was strictly limited to plausible primary constructs within the WISC-V. This parameter restriction was implemented to prevent estimation distortions and avoid potential identification issues during the analyses (Bee et al., 2023). Consistent with the structural constraints inherent to various versions of the WISC-V, several latent factors within the evaluated four- and five-factor model frameworks are underidentified due to being defined by only two subtest indicators. To achieve proper model identification in the hierarchical higher-order CFA models, a standard psychometric protocol was followed by constraining the factor loading of one indicator on each first-order factor and one first-order factor loading on the superordinate higher-order factor to 1 (Brown, 2015; Canivez et al., 2017; Watkins et al., 2017). Conversely, for the specification of two-indicator latent factors within the orthogonal bifactor models, the factor loadings of the subtest pairs on their respective specific group factors were constrained to equality prior to model estimation to ensure proper model identification and stability (Little et al., 1999).

#### 2.3.2 Model fit evaluation

Consistent with best practice, multiple global fit indices were examined to evaluate the structural adequacy of each CFA model solution, adhering to contemporary recommendations for latent factor modeling (Hu and Bentler, 1999; Lai and Green, 2016; McGill et al., 2025). Model fit evaluation was based on a combination of the likelihood-ratio chi-square (*χ*^2^) statistic, the Comparative Fit Index (CFI), the Standardized Root Mean Square Residual (SRMR), the Root Mean Square Error of Approximation (RMSEA) with its 90% confidence interval, and Akaike’s Information Criterion (AIC). Due to the hypersensitivity of the *χ*^2^ test for large sample sizes (Kline, 2016), conventional cut-off criteria were applied. Following common psychometric guidelines, an excellent fit was defined by an RMSEA ≤ 0.050, while acceptable fit thresholds encompassed CFI values ≥ 0.900 alongside an RMSEA ≤ 0.080 (MacCallum et al., 1996; Hu and Bentler, 1999). To comport with stricter criteria for good model fit, a combination of a CFI ≥ 0.950 and an SRMR and RMSEA ≤ 0.060 was utilized (Beribisky and Hancock, 2024). While there are no comparable fixed criteria for information-based indices like the AIC, smaller numerical values were interpreted as capturing a better approximation of the true measurement model after penalizing for parameter complexity (Kaplan, 2000; Marsh and Alamer, 2024).

Because relying blindly on rigid cut-off criteria for global fit evaluation is increasingly criticized in structural equation modeling (McNeish and Wolf, 2023), meaningful statistical margins were utilized to adjudicate between competing CFA model solutions. To demonstrate statistical superiority, a model solution was required to fulfill a combination of strict comparative criteria: it had to exhibit an adequate to good global fit according to the CFI, SRMR, and RMSEA, and demonstrate a substantial incremental improvement. Based on the thresholds established by Chen (2007), this improvement was defined by an increase in CFI of at least 0.010 (ΔCFI ≥ 0.010), a decrease in RMSEA of at least 0.015 (ΔRMSEA ≥ 0.015), and a reduction in AIC of more than 10 (ΔAIC > 10) compared to the less optimal competing models. Beyond these goodness-of-fit statistics, each specified model solution was thoroughly scrutinized for signs of implausible parameters (e.g., negative error variances, Heywood cases, and inadmissible standardized path coefficients).

While a cross-validation design has previously been implemented to explore the primary measurement model and the ten primary subtests on a bifurcated extended standardization sample (Pauls and Daseking, 2021), the five secondary subtests and their specific ancillary index configurations have not yet been subjected to any exploratory factor analyses. Given that the present study represents a genuine, first-time confirmatory evaluation of pre-specified factor model structures proposed by the test publisher (Wechsler, 2014b) and other researchers on a newly configured subsample, the analysis of the full sample size is psychometrically indicated to ensure maximum estimation precision and avoid circularity.

#### 2.3.3 Model-based reliability

Since traditional reliability coefficients are limited when evaluating multifactorial and hierarchical model structures (Yang and Green, 2011; Dunn et al., 2014), model-based reliability analyses were systematically conducted on the model solutions selected based on model evaluation (Pauls and Daseking, 2021; Reise, 2012; Rodriguez et al., 2016; Watkins, 2017). Model-based reliability and replicability coefficients were used to indicate whether the ancillary index scales possess sufficient unique variance to warrant independent clinical interpretation. McDonald’s omega-hierarchical (*ω_H_*) coefficient represents an estimate for the general factor reliability independent of the group factor variances, whereas the omega-hierarchical subscale (*ω_HS_*) indicates the reliability of each group factor that is adjusted for all other group and general factor variances (Brunner et al., 2012). Therefore, *ω_HS_* controls for the proportion of reliability attributable to the general factor and is critical for judging the informative value of each single first-order factor. Robust *ω_HS_* coefficients suggest that most of the specific index variance is independent of the global index, meaning that individual cognitive functioning may be meaningfully interpreted at the specific index level. On the contrary, low *ω_HS_* values suggest that most of the reliable variance of the specific indices is attributable to the global index, which then may compromise the interpretability of index scale scores as unambiguous indicators of specific cognitive domains (Rodriguez et al., 2016). For both *ω_H_* and *ω_HS_*, values near 0.75 should be preferred, whereas values should not be less than 0.50 to warrant distinct interpretation (Reise et al., 2013). Additionally, these omega coefficients were supplemented by the construct replicability coefficient *H* to evaluate how adequately the latent variables were represented by their related indicators (Hancock and Mueller, 2001). Values of *H* should not be less than 0.70 in order to ensure a high quality of indicators and the replicability of latent variables. To provide a multi-perspective evaluation of factor strength, the explained common variance (ECV) was additionally computed as an explicit marker of structural variance specificity (Rodriguez et al., 2016).

Given that subtest scores reflect combinations of higher- and lower-order factors within the hierarchical WISC-V scoring framework, the variance of the second-order factors had to be extracted first to residualize variance proportions from the first-order factors as a necessary prerequisite for estimating these reliability and replicability coefficients (Dombrowski et al., 2018). This way, relative variance apportionment could be examined separately for the first- and second-order factors. Estimating these unique variance proportions then allowed determining how adequately the subtest indicators were represented by their respective factors. The Schmid-Leiman orthogonalization procedure (SL procedure; Schmid and Leiman, 1957) has proven useful in accomplishing such a variance residualization and was systematically applied to the selected best-fitting model solutions prior to model-based reliability analyses. All SL procedures and model-based reliability coefficients were calculated using R (Version 4.6.0; R Core Team, 2026).

## 3 Results

### 3.1 Baseline measurement model and structural integration

CFA results based on maximum likelihood estimation and the corresponding goodness-of-fit statistics for all hierarchical four- and five-factor measurement models analyzed are presented in Table 2. The baseline first-order oblique four-factor model (Baseline 4-factor) yielded an adequate to good fit (CFI = 0.962, SRMR = 0.032, RMSEA = 0.055), while the baseline first-order oblique five-factor model (Baseline 5-factor) achieved a slightly superior fit (CFI = 0.968, SRMR = 0.029, RMSEA = 0.052). The substantial latent correlations among the first-order factors across both model frameworks (Baseline 4-factor: *r* = 0.43 to *r* = 0.85; Baseline 5-factor: *r* = 0.43 to *r* = 0.89) provided the mathematical prerequisite for the extraction of a superordinate second-order general factor *g*. Unlike the findings reported for the US normative data (McGill et al., 2025), all hierarchical four-factor (M1a to M1d) and five-factor (M2a to M2e) models that were specified according to the test publisher (e.g., including *g* as the second-order factor) achieved mathematical convergence. Within the hierarchical four-factor model framework, model solutions exhibited an acceptable to good global fit, with CFI values ranging from 0.956 to 0.966 and RMSEA values between 0.053 and 0.059. Model M1d, which allowed AR to cross-load on the first-order factors *Verbal Comprehension* (VC), *Perceptual Reasoning* (PR), and *Working Memory* (WM), provided the most favorable fit among all hierarchical four-factor model solutions tested (CFI = 0.966, SRMR = 0.030, RMSEA = 0.053). Model M1d also demonstrated statistical improvement over the second-best model M1c, in which AR only loaded on PR and WM but not on VC (ΔCFI = 0.002, ΔRMSEA = 0.001, ΔAIC = 17.279). Within the hierarchical five-factor model framework, the model solutions demonstrated a slightly superior global fit compared to the four-factor model variants. Model M2e (CFI = 0.969, SRMR = 0.030, RMSEA = 0.050), incorporating multi-domain cross-loadings for AR on VC, FR, and WM, and model M2d (CFI = 0.968, SRMR = 0.030, RMSEA = 0.051), in which AR only loaded on VC and WM but not on FR, exhibited the most preferable fit indices among the traditional hierarchical five-factor model solutions that were proposed and analyzed by the test publisher.

**Table 2:** Baseline and hierarchical four- and five-factor CFA models and goodness-of-fit statistics based on the WISC-V primary and secondary subtests for the extended German standardization sample (*N* = 1,466).

CFA model	Indices of model fit								
	*χ* ^2^	*df*	*p*_BS_	*χ*^2^/*df*	CFI	SRMR	RMSEA	90% CI	AIC
Baseline 4-factor	462.79	84	<0.001	5.51	0.962	0.032	0.055	[0.051, 0.060]	564.785
M1a	466.60	86	<0.001	5.43	0.962	0.032	0.055	[0.050, 0.060]	564.595
M1b	520.81	86	<0.001	6.06	0.956	0.033	0.059	[0.054, 0.064]	618.813
M1c	444.26	85	<0.001	5.23	0.964	0.031	0.054	[0.049, 0.059]	544.261
M1d	424.98	84	<0.001	5.06	0.966	0.030	0.053	[0.048, 0.058]	526.982
Baseline 5-factor	393.38	80	<0.001	4.91	0.968	0.029	0.052	[0.047, 0.057]	503.383
M2a	439.84	85	<0.001	5.18	0.964	0.032	0.053	[0.049, 0.058]	539.835
M2b	429.37	85	<0.001	5.05	0.965	0.030	0.053	[0.048, 0.058]	529.374
M2c	403.72	84	<0.001	4.81	0.968	0.030	0.051	[0.046, 0.056]	505.722
M2d	398.96	84	<0.001	4.75	0.968	0.030	0.051	[0.046, 0.056]	500.964
M2e	391.22	83	<0.001	4.71	0.969	0.030	0.050	[0.045, 0.055]	495.220
M2f	328.68	83	<0.001	3.96	0.979	0.026	0.045	[0.040, 0.050]	404.675
**M2f**_**(AWM)**_	**316.32**	**82**	**<0.001**	**3.81**	**0.980**	**0.025**	**0.042**	**[0.037, 0.047]**	**402.319**
M2f _(AWM, QR)_	*Model inadmissible due to negative residual variance for the QR factor*								

Baseline 4-factor = first-order oblique four-factor model with intercorrelated first-order factors, Baseline 5-factor = first-order oblique five-factor model with intercorrelated first-order factors. Hierarchical models M1a to M2e are specified according to the WISC-V Technical and Interpretive Manual and include general intelligence (*g*) as the second-order factor, with Verbal Comprehension (VC), Perceptual Reasoning (PR), Working Memory (WM), and Processing Speed (PS) as first-order factors in all four-factor models, and Verbal Comprehension (VC), Visual Spatial (VS), Fluid Reasoning (FR), Working Memory (WM), and Processing Speed (PS) as first-order factors in all five-factor models. Four-factor models: M1a = Model with SI, VO, IN, and CO loading on VC, BD, VP, MR, and FW loading on PR, AR, DS, PS, and LN loading on WM, and CD, SS, and CA loading on PS. M1b = Model with SI, VO, IN, and CO loading on VC, BD and VP loading on PR, MR, FW, AR, DS, PS, and LN loading on WM, and CD, SS, and CA loading on PS. M1c = Model with SI, VO, IN, and CO loading on VC, BD, VP, MR, FW, and AR loading on PR, AR, DS, PS, and LN loading on WM, and CD, SS, and CA loading on PS. M1d = Model with SI, VO, IN, CO, and AR loading on VC, BD, VP, MR, FW, and AR loading on PR, AR, DS, PS, and LN loading on WM, and CD, SS, and CA loading on PS. Five-factor models: M2a = Model with SI, VO, IN, and CO loading on VC, BD and VP loading on VS, MR and FW loading on FR, AR, DS, PS, and LN loading on WM, and the CD, SS, and CA loading on PS. M2b = Model with SI, VO, IN, and CO loading on VC, BD and VP loading on VS, MR, FW, and AR loading on FR, DS, PS, and LN loading on WM, and CD, SS, and CA loading on PS. M2c = Model with SI, VO, IN, and CO loading on VC, BD and VP loading on VS, MR, FW, and AR loading on FR, AR, DS, PS, and LN loading on WM, and CD, SS, and CA loading on PS. M2d = Model with SI, VO, IN, CO, and AR loading on VC, BD and VP loading on VS, MR and FW loading on FR, AR, DS, PS, and LN loading on WM, and CD, SS, and CA loading on PS. M2e = Model with SI, VO, IN, CO, and AR loading on VC, BD and VP loading on VS, MR, FW and AR loading on FR, AR, DS, PS, and LN loading on WM, and CD, SS, and CA loading on PS. M2f = modified M2a with an additional correlation between FR and VS and a direct loading of AR on *g*. M2f _(AWM)_ = expanded M2f with DS and LN loading on Auditory Working Memory (AWM) as a minor factor nested within WM. M2f _(AWM, QR)_ = expanded M2f _(AWM)_ with FW and AR loading on Quantitative Reasoning (QR) as a minor factor nested within FR. *p*_BS_ = *p*-value derived from the Bollen–Stine bootstrapping procedure with 2,000 resamples. CFI, Comparative Fit Index; SRMR, Standardized Root Mean Square Residual; RMSEA, Root Mean Square Error of Approximation; 90% CI, Confidence interval for RMSEA; AIC, Akaike's Information Criterion. Values for the best-fitting model are indicated in bold.

Goodness-of-fit statistics for the alternative bifactor four- and five-factor models analyzed are presented in Table 3. All four-factor (M1a* to M1d*) and five-factor (M2a* to M2e*) model solutions that were adapted from the test publisher’s traditional hierarchical models demonstrated a markedly superior global fit compared to their hierarchical model counterparts M1a to M1d and M2a to M2e. Within the four-factor model framework, CFI values ranged between 0.977 and 0.979, while RMSEA values remained highly stable at values from 0.044 to 0.046. Among these four-factor model solutions, M1c* (CFI = 0.979, SRMR = 0.025, RMSEA = 0.044), featuring cross-loadings of AR on the group factors PR and WM, and M1d* (CFI = 0.979, SRMR = 0.024, RMSEA = 0.044), featuring an additional loading of AR on VC, demonstrated the best fit to the data. Within the five-factor model framework, bifactor model solutions demonstrated equally robust fit indices. Model M2d* (CFI = 0.977, SRMR = 0.026, RMSEA = 0.045), incorporating AR loadings on VC and WM, and model M2e* (CFI = 0.977, SRMR = 0.026, RMSEA = 0.046), featuring an additional AR loading on FR, exhibited the most favorable fit among those adapted from the test publisher’s traditional hierarchical five-factor model solutions.

**Table 3:** Bifactor four- and five factor CFA models and goodness-of-fit statistics based on the WISC-V primary and secondary subtests for the extended German standardization sample (*N* = 1,466).

CFA model	Indices of model fit								
	*χ* ^2^	*df*	*p*_BS_	*χ*^2^/*df*	CFI	SRMR	RMSEA	90% CI	AIC
M1a*	290.50	75	<0.001	3.87	0.978	0.025	0.044	[0.039, 0.050]	410.501
M1b*	310.12	76	<0.001	4.04	0.977	0.026	0.046	[0.040, 0.051]	425.117
M1c*	284.70	74	<0.001	3.85	0.979	0.025	0.044	[0.039, 0.050]	406.699
M1d*	283.34	73	<0.001	3.88	0.979	0.024	0.044	[0.039, 0.050]	407.338
M2a*	304.88	77	<0.001	3.96	0.977	0.026	0.045	[0.040, 0.050]	420.880
M2b*	327.94	76	<0.001	4.32	0.975	0.026	0.048	[0.042, 0.053]	445.936
M2c*	303.14	75	<0.001	4.04	0.977	0.026	0.046	[0.040, 0.051]	423.136
M2d*	302.19	76	<0.001	3.98	0.977	0.026	0.045	[0.040, 0.050]	420.188
M2e*	301.77	74	<0.001	4.08	0.977	0.026	0.046	[0.041, 0.051]	423.766
M2f*	**287.20**	**76**	**<0.001**	**3.78**	**0.979**	**0.025**	**0.044**	**[0.038, 0.049]**	**405.198**
M2f* _(AWM)_	*Model failed to converge, number of iterations exceeded*								
M2f* _(AWM, QR)_	*Model failed to converge, number of iterations exceeded*								

Bifactor models M1a* to M2e* are specified according to the hierarchical models reported in the WISC-V *Technical and Interpretive Manual* and include general intelligence (*g*) as the general factor, with Verbal Comprehension (VC), Perceptual Reasoning (PR), Working Memory (WM), and Processing Speed (PS) as group factors in all four-factor models, and Verbal Comprehension (VC), Visual Spatial (VS), Fluid Reasoning (FR), Working Memory (WM), and Processing Speed (PS) as group factors in all five-factor models. Four-factor models: M1a* = Model with SI, VO, IN, and CO loading on VC, BD, VP, MR, and FW loading on PR, AR, DS, PS, and LN loading on WM, and CD, SS, and CA loading on PS. M1b* = Model with SI, VO, IN, and CO loading on VC, BD and VP loading on PR, MR, FW, AR, DS, PS, and LN loading on WM, and CD, SS, and CA loading on PS. M1c* = Model with SI, VO, IN, and CO loading on VC, BD, VP, MR, FW, and AR loading on PR, AR, DS, PS, and LN loading on WM, and CD, SS, and CA loading on PS. M1d* = Model with SI, VO, IN, CO, and AR loading on VC, BD, VP, MR, FW, and AR loading on PR, AR, DS, PS, and LN loading on WM, and CD, SS, and CA loading on PS. Five-factor models: M2a* = Model with SI, VO, IN, and CO loading on VC, BD and VP loading on VS, MR and FW loading on FR, AR, DS, PS, and LN loading on WM, and the CD, SS, and CA loading on PS. M2b* = Model with SI, VO, IN, and CO loading on VC, BD and VP loading on VS, MR, FW, and AR loading on FR, DS, PS, and LN loading on WM, and CD, SS, and CA loading on PS. M2c* = Model with SI, VO, IN, and CO loading on VC, BD and VP loading on VS, MR, FW, and AR loading on FR, AR, DS, PS, and LN loading on WM, and CD, SS, and CA loading on PS. M2d* = Model with SI, VO, IN, CO, and AR loading on VC, BD and VP loading on VS, MR and FW loading on FR, AR, DS, PS, and LN loading on WM, and CD, SS, and CA loading on PS. M2e* = Model with SI, VO, IN, CO, and AR loading on VC, BD and VP loading on VS, MR, FW and AR loading on FR, AR, DS, PS, and LN loading on WM, and CD, SS, and CA loading on PS. M2f* = modified M2a* with an additional correlation between FR and VS. M2f* _(AWM)_ = expanded M2f* with DS and LN loading on Auditory Working Memory (AWM) as a minor factor nested within WM. M2f* _(AWM, QR)_ = expanded M2f* _(AWM)_ with FW and AR loading on Quantitative Reasoning (QR) as a minor factor nested within FR. *p*_BS_ = *p*-value derived from the Bollen–Stine bootstrapping procedure with 2,000 resamples. CFI, Comparative Fit Index; SRMR, Standardized Root Mean Square Residual; RMSEA, Root Mean Square Error of Approximation; 90% CI, Confidence interval for RMSEA; AIC, Akaike's Information Criterion. Values for the best-fitting model are indicated in bold.

In addition to the traditional measurement models described above, an alternative five-factor model solution proposed by Reynolds and Keith (2017) was evaluated as well. This hierarchical CFA model (M2f), containing a correlated residual path between FR and VS alongside a direct loading of AR on *g*, demonstrated a substantially superior model fit over all other hierarchical four- and five-factor model solutions analyzed (CFI = 0.979, SRMR = 0.026, RMSEA = 0.045). Compared to the best-fitting model proposed by the test publisher (M2e), model M2f provided a notable incremental fit improvement (ΔCFI = 0.010, ΔRMSEA = 0.005, ΔAIC = 90.545). The bifactor model counterpart M2f* exhibited an equally exceptional and robust fit to the data (CFI = 0.979, SRMR = 0.025, RMSEA = 0.044). When considering all traditional and alternative model solutions analyzed across both hierarchical and bifactor model frameworks, the hierarchical model M2f and the bifactor model M2f* demonstrated the overall superior and most favorable fit to the data. Consequently, M2f and M2f* were selected as the baseline measurement models from which to incrementally add the hypothesized factors associated with the ancillary indices *Auditory Working Memory* (AWM) and *Quantitative Reasoning* (QR) as part of an expanded WISC-V measurement model.

**Figure 1:**
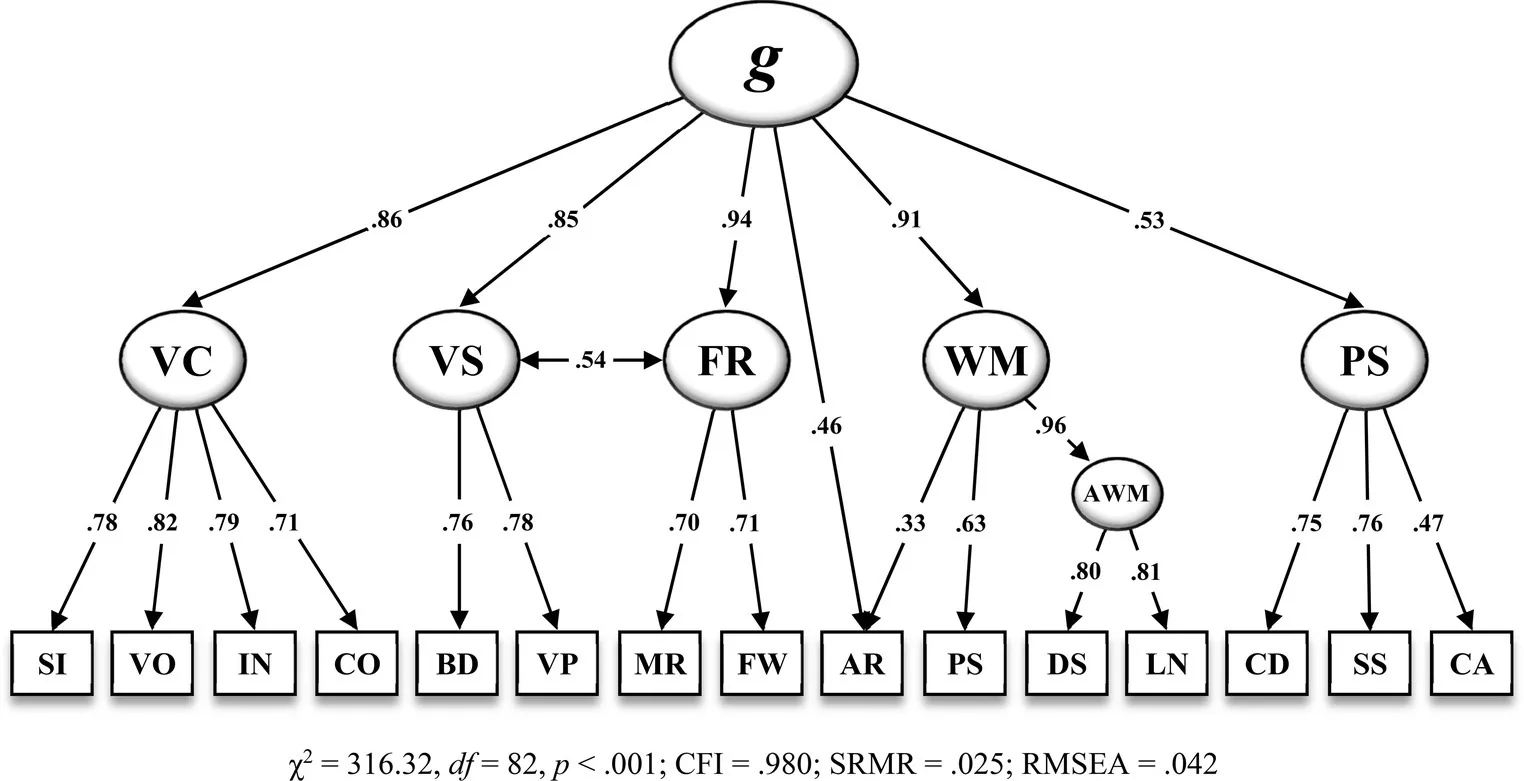
Second-order five-factor model including standardized estimations for the extended German standardization sample (*N* = 1,466) on the WISC-V primary and ancillary subtests (M2f (AWM) in Table 2). Subtests: SI, Similarities; VO, Vocabulary; IN, Information; CO, Comprehension; BD, Block Design; VP, Visual Puzzles; MR, Matrix Reasoning; FW, Figure Weights; AR, Arithmetic; PS, Picture Span; DS, Digit Span; LN, Letter-Number Sequencing; CD, Coding; SS, Symbol Search; CA, Cancellation. First-order factors: VC, Verbal Comprehension; VS, Visual Spatial; FR, Fluid Reasoning; WM, Working Memory; PS, Processing Speed. Second-order factor: *g*, general intelligence; CFI, Comparative Fit Index; SRMR, Standardized Root Mean Square Residual; RMSEA, Root Mean Square Error of Approximation. All standardized parameter estimates are significant at *p* < 0.001. Residual parameters are omitted for clarity.

In the hierarchical model framework, the integration of AWM as a minor factor nested within WM in model M2f _(AWM)_ yielded a good fit to the data (CFI = 0.980, SRMR = 0.025, RMSEA = 0.042) and at least a slight descriptive improvement compared to the corresponding baseline measurement model (ΔCFI = 0.001, ΔRMSEA = 0.003, ΔAIC = 2.356). While global fit indices were nearly identical to those obtained from the baseline measurement model M2f, the lower AIC value for M2f _(AWM)_ indicated that it should be judged to fit the data better in comparison to other nested configurations (Vrieze, 2012). As depicted in Figure 1, AWM appears to emerge as a psychometrically plausible construct and subdomain of WM, mirroring the findings reported for the US normative sample (McGill et al., 2025). Conversely, a critical psychometric boundary was reached when additionally attempting to integrate QR as a minor factor nested within FR in model M2f _(AWM, QR)_. This expanded model solution, incorporating both minor factors for AWM and QR, turned out to be structurally inadmissible due to a severe Heywood case manifested as a negative residual variance for the QR factor. In the bifactor model framework, the corresponding model solutions, integrating AWM as a minor factor nested within WM in model M2f* _(AWM)_ and subsequently integrating QR as a minor factor nested within FR in model M2f _(AWM, QR)_, both failed to achieve mathematical convergence. While AWM retained enough specific variance to function as a narrow subordinate dimension in the hierarchical model framework, the QR completely failed to maintain structural integrity or functional independence across both hierarchical and bifactor model frameworks.

As depicted in Table 4, model-based reliability analyses on the model M2f _(AWM)_ showed that smaller proportions of explained common variance, ranging from ECV = 0.013 (AWM) to ECV = 0.119 (PS), were uniquely associated with the specific group factors than those associated with *g* (ECV = 0.698). Conclusively, about 69.8% of the common variance in the subtest indicators appeared to be uniquely explained by the superordinate general factor. *ω_HS_* coefficient values for the specific group factors, ranging from *ω_HS_* = 0.065 (AWM) to *ω_HS_* = 0.507 (PS), were rather small when compared to the *ω_H_* coefficient value for *g* (*ω_H_* = 0.850). While the general factor turned out to be precisely measured by the subtest indicators, almost all *ω_HS_* coefficient values for the group factors fell short of the required minimum criterion of 0.500, with the sole exception of PS. Specifically, the nested group factor for Auditory Working Memory (AWM) yielded the lowest estimates. Furthermore, while the construct replicability coefficient *H* suggested that *g* was well defined by the subtest indicators (*H* = 0.912), none of the coefficient values for the group factors, ranging from *H* = 0.101 (AWM) to *H* = 0.614 (PS), met the recommended threshold of 0.700. This indicated that the group factors are not adequately defined by their indicators to produce consistent scores across measurements independent of *g*.

**Table 4:** Sources of variance in the WISC-V primary and secondary subtests for the extended German WISC-V standardization sample (*N* = 1,466) according to the SL orthogonalized second-order five-factor model (M2f (AWM) in Table 2).

WISC-V subtest	*g*		VC		VS		FR		WM		AWM		PS		*h* ^2^	*u* ^2^
	*b*	*S* ^2^	*b*	*S* ^2^	*b*	*S* ^2^	*b*	*S* ^2^	*b*	*S* ^2^	*b*	*S* ^2^	*b*	*S* ^2^		
SI	0.670	0.449	0.407	0.165											0.615	0.385
VC	0.699	0.488	0.424	0.180											0.667	0.333
IN	0.671	0.450	0.407	0.166											0.616	0.384
CO	0.605	0.366	0.367	0.135											0.501	0.499
BD	0.646	0.417			0.402	0.162									0.579	0.421
VP	0.661	0.436			0.411	0.169									0.605	0.395
MR	0.660	0.436					0.250	0.063							0.498	0.502
FW	0.660	0.436					0.250	0.063							0.498	0.502
AR	0.752	0.566							0.137	0.019					0.584	0.416
PS	0.568	0.322							0.262	0.069					0.391	0.609
DS	0.692	0.479							0.319	0.102	0.228	0.052			0.634	0.366
LN	0.708	0.501							0.327	0.107	0.233	0.054			0.663	0.337
CD	0.402	0.162											0.640	0.410	0.572	0.428
SS	0.405	0.164											0.644	0.415	0.579	0.421
CA	0.248	0.061											0.395	0.156	0.217	0.783
Total *S*^2^		0.382		0.161		0.165		0.063		0.074		0.053		0.327	0.548	0.452
ECV		0.698		0.079		0.040		0.015		0.036		0.013		0.119		
*ω*		0.930		0.857		0.744		0.665		0.834		0.786		0.707		
*ω_H_*/*ω_HS_*		0.850		0.230		0.208		0.084		0.105		0.065		0.507		
Relative *ω*		0.819		0.269		0.279		0.126		0.125		0.082		0.717		
*H*		0.912		0.435		0.284		0.118		0.246		0.101		0.614		
PUC		0.819														

General factor: *g* = General Intelligence. Group factors: VC, Verbal Comprehension; VS, Visual Spatial; FR, Fluid Reasoning; WM, Working Memory; AWM, Auditory Working Memory; PS, Processing Speed. Subtest indicators: SI, Similarities; VO, Vocabulary; IN, Information; CO, Comprehension; BD, Block Design; VP, Visual Puzzles; MR, Matrix Reasoning; FW, Figure Weights; AR, Arithmetic; PS, Picture Span; DS, Digit Span; LN, Letter-Number Sequencing; CD, Coding; SS, Symbol Search; CA, Cancellation. *b* = factor loading (significant at *p* < 0.05), *S*^2^ = explained variance, *h*^2^ = communality, *u*^2^ = unique variance, ECV = explained common variance, *ω* = omega coefficient, *ω_H_* = omega-hierarchical coefficient (general factor), *ω_HS_* = omega-hierarchical coefficient (group factors), Relative *ω* = relative omega coefficient (proportion of reliable variance attributable to a specific factor), *H* = replicability index (construct reliability), PUC = percentage of uncontaminated correlations.

### 3.2 Structural composition of nonverbal ability

**Figure 2:**
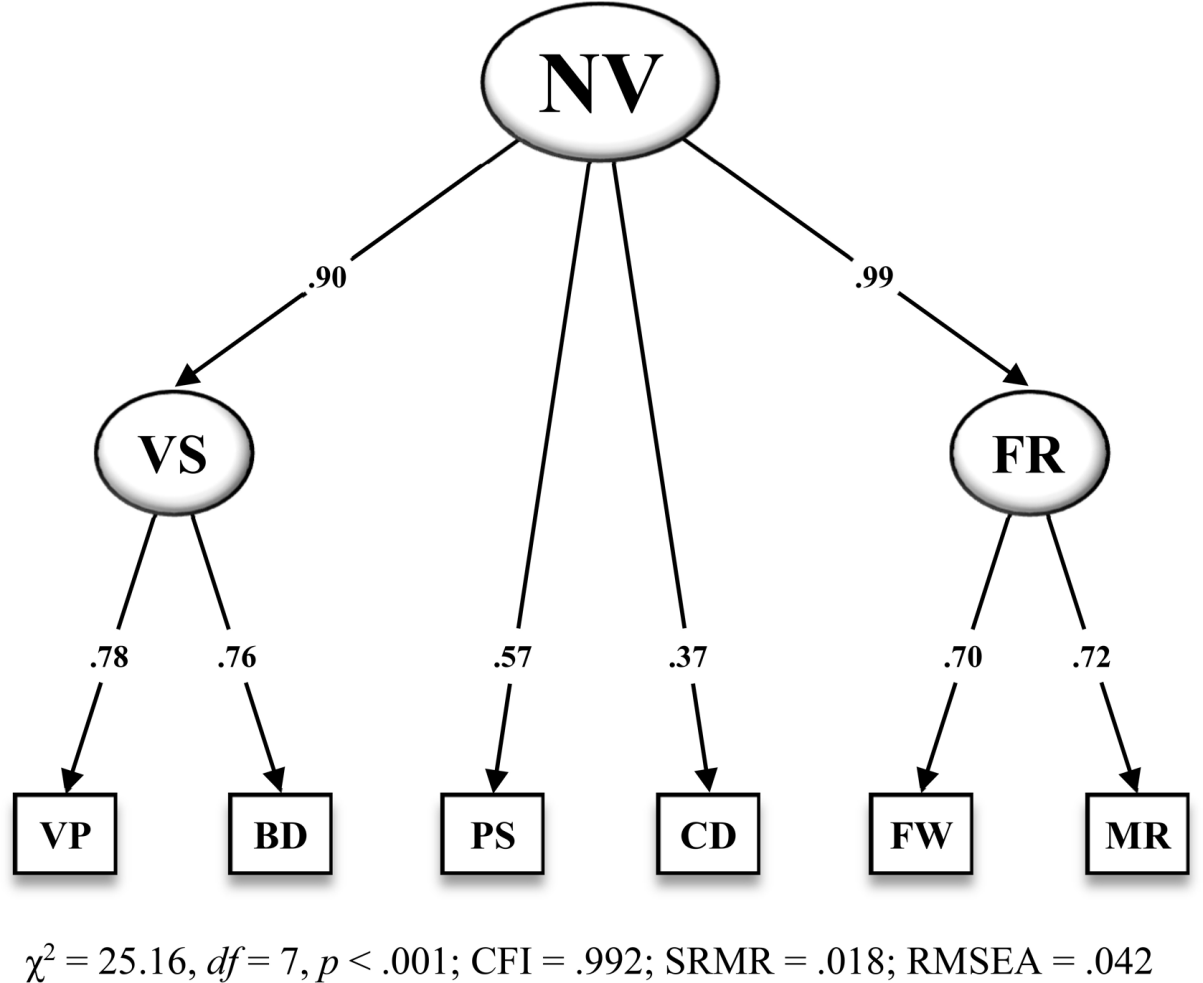
Second-order two-factor model including standardized estimations for the extended German standardization sample (*N* = 1,466) on the WISC-V primary subtests for the Nonverbal Index (M3b in Table 5). Subtests: VP, Visual Puzzles; BD, Block Design; PS, Picture Span; CD, Coding; FW, Figure Weights; MR, Matrix Reasoning. First-order factors: VS, Visual Spatial; FR, Fluid Reasoning. Second-order factor: NV, Nonverbal ability; CFI, Comparative Fit Index; SRMR, Standardized Root Mean Square Residual; RMSEA, Root Mean Square Error of Approximation. All standardized parameter estimates are significant at *p* < 0.001. Residual parameters are omitted for clarity.

Separate CFAs were conducted to determine whether the nonverbal subtests of the German WISC-V reflect a unified, single latent nonverbal ability factor (NV) for the *Nonverbal Index* (NVI) or a multidimensional, hierarchical construct. As indicated by the goodness-of-fit statistics presented in the top section of Table 5, model fit improved significantly from a global and undifferentiated to a more differentiated hierarchical model structure. Although the one-factor model solution M3a, with MR, FW, CD, PS, BD, and VP loading on the single general factor NV, demonstrated an acceptable fit to the data (CFI = 0.978, SRMR = 0.026, RMSEA = 0.063), the second-order two-factor model M3b, which integrates FR and VS as intermediate first-order factors, achieved a better global fit (CFI = 0.992, SRMR = 0.018, RMSEA = 0.042). A comparison between both model solutions revealed a substantial incremental fit improvement of M3b over M3a (ΔCFI = 0.014, ΔRMSEA = 0.021, ΔAIC = 32.119), confirming the empirical superiority of the hierarchical second-order two-factor model solution. Consequently, this second-order model was retained as the final structural representation of the NVI (see Figure 2).

**Table 5:** CFA models and goodness-of-fit statistics based on the WISC-V primary subtests for the ancillary indices NVI, GAI, and CPI for the extended German standardization sample (*N* = 1,466).

CFA model	Indices of model fit								
	*χ* ^2^	*df*	*p*	*χ*^2^/*df*	CFI	SRMR	RMSEA	90% CI	AIC
NVI:									
M3a	61.38	9	<0.001	6.82	0.978	0.026	0.063	[0.049, 0.078]	97.281
**M3b**	**25.16**	**7**	**<0.001**	**3.60**	**0.992**	**0.018**	**0.042**	**[0.025, 0.060]**	**65.162**
GAI and CPI:									
M4a	1345.84	27	<0.001	49.85	0.713	0.244	0.183	[0.174, 0.191]	1399.835
M4b	598.75	26	<0.001	23.03	0.875	0.066	0.123	[0.114, 0.131]	654.748
M4c	967.77	24	<0.001	40.32	0.794	0.239	0.164	[0.155, 0.173]	1027.769
**M4d**	**67.63**	**22**	**<0.001**	**3.07**	**0.990**	**0.020**	**0.038**	**[0.028, 0.048]**	**131.634**
M4e	127.69	31	<0.001	4.12	0.984	0.023	0.046	[0.038, 0.055]	195.687

NVI, Nonverbal Index; GAI, General Ability Index; CPI, Cognitive Processing Index. M3a = One-factor model with MR, FW, CD, PS, BD, and VP loading on the single factor Nonverbal (NV). M3b = Second-order two-factor model with MR and FW loading on the first-order factor Fluid Reasoning (FR), BD and VP loading on the first-order factor Visual Spatial (VS), and CD, PS, FS, and VS loading on the second-order factor NV. M4a = First-order two-factor orthogonal model with SI, VO, BD, FW, and MR loading on the first-order factor General Ability (GA), and DS, PS, CD, and SS loading on the first-order factor Cognitive Processing (CP). M4b = First-order two-factor oblique model with the same specification as M4a, except for a correlation between GA and CP. M4c = Second-order four-factor orthogonal model with SI and VO loading on the first-order factor VC, FW and MR loading on the first-order factor FR, DS and PS loading on the first-order factor WM, CD and SS loading on the first-order factor PS, BD, VS, and FS loading on the second-order factor GA, and WM and PS loading on the second-order factor CP. M4d = Second-order four-factor oblique model with the same specification as M4c, except for a correlation between GA and CP. M4e = Third-order four-factor model with SI, VO, and IN loading on the first-order factor Verbal Comprehension (VC), BD, VP, and MR loading on the first-order factor Perceptual Reasoning (PR), DS and AR loading on the first-order factor Working Memory (WM), CD and SS loading on the first-order factor Processing Speed (PS), the first-order factors VC and PR loading on the second-order factor GA, the first-order factors WM and PS loading on the second-order factor CP, and the second-order factors GA and CP loading on the third-order factor General Intelligence (*g*). CFI, Comparative Fit Index; SRMR, Standardized Root Mean Square Residual; RMSEA, Root Mean Square Error of Approximation; 90% CI, Confidence interval for RMSEA; AIC, Akaike's Information Criterion. Values for the best-fitting model are indicated in bold.

Results of the model-based reliability analyses on model M3b are summarized in Table 6. Again, unique variance proportions captured by the specific group factors were remarkably small, with the explained common variance ranging from ECV = 0.006 (FR) to ECV = 0.087 (VS). In contrast, the superordinate NV factor emerged as highly dominant, accounting uniquely for approximately 90.7% of the shared variance among the subtests (ECV = 0.907). Reflecting this uneven variance allocation, the *ω_HS_* coefficient values for the specific group factors turned out to be small, with *ω_HS_* = 0.011 for FR and *ω_HS_* = 0.144 for VS, particularly when contrasted with the robust hierarchical reliability of the general NV factor (*ω_H_* = 0.783). Although the global nonverbal ability dimension appeared to be well-saturated by its underlying indicators, both group factors fell drastically below the required minimum criterion of 0.500. While the construct replicability index confirmed that the NV factor was solidly defined by its indicators across measurements (*H* = 0.815), the corresponding coefficients for the group factors, with *H* = 0.016 for FR and *H* = 0.206 for VS, remained far below the established 0.700 threshold.

**Table 6:** Sources of variance in the WISC-V primary subtests for the Nonverbal Index for the extended German WISC-V standardization sample (*N* = 1,466) according to the SL orthogonalized second-order two-factor model (M3b in Table 5).

WISC-V subtest	NV		VS		FR		*h* ^2^	*u* ^2^
	*b*	*S* ^2^	*b*	*S* ^2^	*b*	*S* ^2^		
VP	0.703	0.494	0.345	0.119			0.613	0.387
BD	0.679	0.461	0.333	0.111			0.572	0.428
PS	0.572	0.327					0.327	0.673
CD	0.367	0.135					0.135	0.865
FW	0.690	0.477			0.088	0.008	0.484	0.516
MR	0.711	0.506			0.091	0.008	0.514	0.486
Total *S*^2^		0.400		0.115		0.008	0.441	0.559
ECV		0.907		0.087		0.006		
*ω*		0.810		0.744		0.666		
*ω_H_*/*ω_HS_*		0.783		0.144		0.011		
Relative *ω*		0.867		0.194		0.016		
*H*		0.815		0.206		0.016		
PUC		0.867						

General factor: NV, Nonverbal Ability. Group factors: VS, Visual Spatial; FR, Fluid Reasoning. Subtest indicators: VP, Visual Puzzles; BD, Block Design; PS, Picture Span; CD, Coding; FW, Figure Weights; MR, Matrix Reasoning. *b* = factor loading (significant at *p* < 0.05), *S*^2^ = explained variance, *h*^2^ = communality, *u*^2^ = unique variance, ECV = explained common variance, *ω* = omega coefficient, *ω_H_* = omega-hierarchical coefficient (general factor), *ω_HS_* = omega-hierarchical coefficient (group factors), Relative *ω* = relative omega coefficient (proportion of reliable variance attributable to a specific factor), *H* = replicability index (construct reliability), PUC = percentage of uncontaminated correlations.

### 3.3 Structural composition of general ability and cognitive proficiency

An additional series of independent CFAs was executed to examine whether the latent dimensions for general ability (GA) and cognitive proficiency (CP), reflecting the *General Ability Index* (GAI) and the *Cognitive Proficiency Index* (CPI), function as independent and standalone dimensions or as intermediary dimensions situated within a broader hierarchical model framework. As shown in the bottom section of Table 5, the goodness-of-fit statistics for the two-factor model solutions framing the factors GA and CP as first-order orthogonal (M4a) or first-order oblique (M4b) factors, as well as the second-order four-factor orthogonal model M4c featuring GA and CP as second-order factors, were unequivocally inadequate, failing to meet standard criteria for acceptable model fit. Among the alternative model solutions evaluated, the second-order four-factor oblique model M4d—wherein the second-order factors GA and CP are allowed to correlate and BD loads directly onto the GA factor—provided an excellent fit to the data (CFI = 0.990, SRMR = 0.020, RMSEA = 0.038).

**Figure 3:**
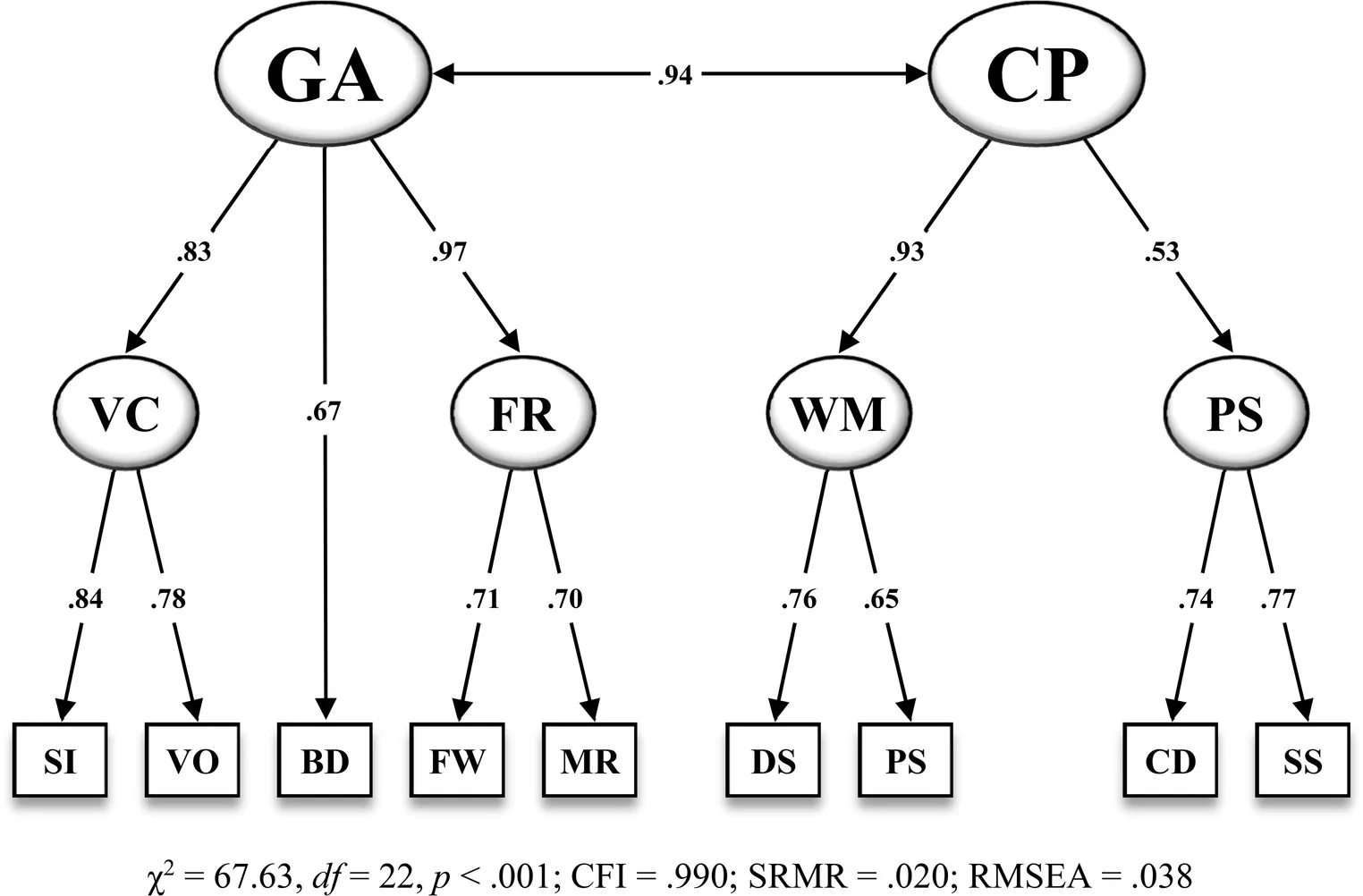
Second-order four-factor oblique model including standardized estimations for the extended German standardization sample (*N* = 1,466) on the WISC-V primary subtests for the General Ability and Cognitive Proficiency Indices (M4d in Table 5). Subtests: SI, Similarities; VO, Vocabulary; BD, Block Design; FW, Figure Weights; MR, Matrix Reasoning; DS, Digit Span; PS, Picture Span; CD, Coding; SS, Symbol Search. First-order factors: VC, Verbal Comprehension; FR, Fluid Reasoning; WM, Working Memory; PS, Processing Speed. Second-order factors: GA, General ability; CP, Cognitive Proficiency; CFI, Comparative Fit Index; SRMR, Standardized Root Mean Square Residual; RMSEA, Root Mean Square Error of Approximation. All standardized parameter estimates are significant at *p* < 0.001. Residual parameters are omitted for clarity.

Unlike the findings reported by McGill et al. (2025) where the alternative hierarchical model solution based on Zhu (2009) could not be evaluated due to a Heywood case, the corresponding third-order four-factor model M4e achieved flawless mathematical convergence within the present analyses on the extended German standardization data (CFI = 0.984, SRMR = 0.023, RMSEA = 0.046). However, a direct comparison between the two valid model solutions M4e and M4d revealed that the complex third-order model framework was statistically outperformed by the second-order oblique model solution (ΔCFI = 0.006, ΔRMSEA = 0.008, ΔAIC = 64.053). Therefore, model M4d was retained as the final and optimal structural representation of the GAI and CPI (see Figure 3).

Model-based reliability coefficients for model M4d are detailed in Table 7. With the explained common variance ranging from ECV = 0.012 (FR) to ECV = 0.169 (PS), specific group factors were found to explain substantially smaller proportions of common variance than the superordinate GA (ECV = 0.472) and CP (ECV = 0.240) factors. When compared with the hierarchical reliability coefficients of GA (*ω_H_* = 0.787) and CP (*ω_H_* = 0.540), reliability for most of the group factors turned out to be rather weak, ranging from *ω_HS_* = 0.038 (FR) to *ω_HS_* = 0.527 (PS). Except for PS, every single group factor fell markedly short of the required minimum criterion of 0.500. While construct replicability coefficients indicated that the superordinate GA factor was robustly defined across measurements (*H* = 0.813), the coefficients for the superordinate CP factor (*H* = 0.659) and all specific group factors, ranging from *H* = 0.055 (FR) to *H* = 0.587 (PS), failed to meet the recommended 0.700 threshold. Conclusively, CP and all group factors failed to be sufficiently defined by their indicators to warrant stable and consistent index scale scores.

**Table 7:** Sources of variance in the WISC-V primary subtests for the General Ability and the Cognitive Proficiency Indices for the extended German WISC-V standardization sample (*N* = 1,466) according to the SL orthogonalized second-order four-factor oblique model (M4d in Table 5).

WISC-V subtest	GA		CP		VC		FR		WM		PS		*h* ^2^	*u* ^2^
	*b*	*S* ^2^	*b*	*S* ^2^	*b*	*S* ^2^	*b*	*S* ^2^	*b*	*S* ^2^	*b*	*S* ^2^		
SI	0.697	0.486			0.461	0.213							0.699	0.301
VC	0.649	0.421			0.429	0.184							0.605	0.395
BD	0.686	0.471											0.471	0.529
FW	0.686	0.471					0.169	0.029					0.500	0.500
MR	0.685	0.469					0.169	0.028					0.497	0.503
DS			0.705	0.496					0.274	0.075			0.572	0.428
PS			0.601	0.361					0.234	0.055			0.416	0.584
CD			0.393	0.154							0.632	0.399	0.554	0.446
SS			0.408	0.167							0.656	0.431	0.598	0.402
Total *S*^2^		0.464		0.295		0.199		0.028		0.065		0.415	0.546	0.454
ECV		0.472		0.240		0.081		0.012		0.026		0.169		
*ω*		0.849		0.773		0.789		0.665		0.660		0.731		
*ω_H_*/*ω_HS_*		0.787		0.540		0.240		0.038		0.087		0.527		
Relative *ω*		0.927		0.698		0.304		0.057		0.131		0.721		
*H*		0.813		0.659		0.332		0.055		0.122		0.587		
PUC		0.889		0.889										

General factors: GA, General Ability; CP, Cognitive Proficiency. Group factors: VC, Verbal Comprehension; FR, Fluid Reasoning; WM, Working Memory; PS, Processing Speed. Subtest indicators: SI, Similarities; VO, Vocabulary; BD, Block Design; FW, Figure Weights; MR, Matrix Reasoning; DS, Digit Span; PS, Picture Span; CD, Coding; SS, Symbol Search. *b* = factor loading (significant at *p* < 0.05), *S*^2^ = explained variance, *h*^2^ = communality, *u*^2^ = unique variance, ECV = explained common variance, *ω* = omega coefficient, *ωH* = omega-hierarchical coefficient (general factor), *ω_HS_* = omega-hierarchical coefficient (group factors), Relative *ω* = relative omega coefficient (proportion of reliable variance attributable to a specific factor), *H* = replicability index (construct reliability), PUC = percentage of uncontaminated correlations.

## 4 Discussion

The development of the Wechsler scales has historically favored the proliferation of index scale composites, yet this inflation of interpretive scores has rarely been accompanied by fundamental shifts in the actual subtest composition (Frazier and Youngstrom, 2007). While the primary hierarchical five-factor model of the WISC-V is supported by independent structural research to some extent, a profound empirical vacuum remains regarding the ancillary index scales. Neither the WISC-V *Technical and Interpretive Manual* nor other clinical manuals offer structural verification for these ancillary index scales, leaving their statistical necessity largely unestablished. Although details for the practical administration and calculation of these scales are provided by interpretive literature (e.g., Kaufman et al., 2016; Weiss et al., 2016), the latter fails to provide the factor-analytical evidence required to substantiate their diagnostic application. This lack of structural validation leaves clinicians in a difficult position, as they are encouraged to utilize index scores that lack a solid empirical and psychometric foundation for daily decision-making (Beaujean and Benson, 2019). This uncertainty is further amplified by a long historical pattern of failed empirical replications regarding complex subtest profile analysis within the Wechsler tradition (Smith and Watkins, 2004; Styck et al., 2019). Furthermore, the degree to which ancillary index scales represent unique cognitive dimensions—rather than minor statistical artifacts heavily saturated by *g*—remains a highly contentious issue regarding their true incremental validity beyond the FSIQ.

McGill et al. (2025) conducted a rigorous factor-analytical examination of the US normative data, demonstrating that the structural validity of some ancillary index scales is substantially more fragile than what is assumed given their extensive use in clinical practice. Determining whether such psychometric properties generalize to the German WISC-V represents a critical milestone for evidence-based practice. By executing a series of systematic hierarchical and bifactor CFA models on the extended German standardization sample, the present study directly addresses this research question. In doing so, our structural findings generally align with the baseline configurations reported by McGill et al. (2025), suggesting a robust overarching architecture across international adaptations. Beyond mere replication, however, our integration of bifactor modeling and model-based reliability estimation provides a more critical appraisal. While global fit indices might suggest acceptable multidimensionality, model-based reliability indices additionally reveal that the specific group factors capture limited unique variance once a general factor (e.g., *g*) is systematically residualized, thus providing the necessary empirical criteria to evaluate whether the ancillary scales possess sufficient statistical autonomy for clinical application. To delineate the psychometric limitations and clinical interpretability established by the present factor-analytical approach, the structural validity and diagnostic viability of each specific ancillary index scale are systematically reviewed below.

### 4.1 Structural validity of the Auditory Working Memory Index (AWM)

Regarding the first objective of the present study, results of the current CFAs provide evidence for an independent dimension representing the *Auditory Working Memory Index* (AWM). Expanding the primary five-factor measurement model of the German WISC-V to include a latent factor for AWM yielded a mathematically stable parameter estimation with excellent global fit criteria. The specific subtest indicators Digit Span (DS) and Letter-Number Sequencing (LN) exhibited high, statistically significant factor loadings on this newly specified group factor, indicating that the underlying AWM dimension is structurally identifiable within the German standardization sample.

However, parameter estimations for the expanded primary five-factor measurement model M2f _(AWM)_ introduce substantial theoretical ambiguity regarding what AWM might actually reflect. As indicated by the loading of AWM on WM of 0.96 (see Figure 1), this extreme collinearity suggests that AWM might not account for sufficient unique variance in the associated subtest indicators beyond WM. This structural redundancy is further substantiated by the model-based reliability and replicability analyses. Once the variance of WM is statistically controlled, AWM fails to retain sufficient unique, reliable variance and thus lacks structural stability. This severe depletion suggests that the ancillary index scale score for AWM is almost entirely saturated by the underlying working memory dimension rather than reflecting an independent, autonomous memory dimension. Consequently, diagnostic inferences or clinical decisions derived from index scale score discrepancies between the primary WMI and the ancillary AWM are highly precarious and psychometrically specious. From a historical viewpoint, this structural redundancy is deeply rooted in the evolution of the Wechsler scales. Because the AWM is derived from the exact subtest dyad (DS and LN) that originally defined the WMI in the WISC-IV, its modern reclassification as a narrower, ancillary index scale suggests that earlier Wechsler taxonomies may have mislabeled a subordinate cognitive facet as a broader, higher-order construct. Such a misalignment has led to several competing interpretations within the literature regarding the exact cognitive natures of AWM and the WMI (see McGill et al., 2025, for an overview). Furthermore, the task hierarchy within the WISC-V further intensifies these interpretive concerns. Independent factor-analytical studies have consistently demonstrated that LN serves as a superior indicator of working memory capacity compared to PS (e.g., Canivez et al., 2016, 2017). Despite this established empirical superiority, however, LN has been relegated to a secondary subtest to accommodate the primary subtest PS within the WISC-V scoring framework.

Structural modifications and shifting subtest configurations across test revisions may severely threaten the validity of longitudinal cognitive profile analyses, as these changes violate the basic assumptions for measurement invariance and test equating between test versions such as the WISC-IV and WISC-V (Pendergast et al., 2017). As indicated by a relatively weak correlation for the WMI across the WISC-IV and WISC-V (*r* = 0.59) reported in the German WISC-V *Technical and Interpretive Manual*, the two test versions might capture different aspects of the same underlying cognitive domain. Conclusively, direct comparisons between the corresponding index scores—despite implying to reflect the same cognitive dimension—are highly problematic (Beaujean et al., 2018). These discrepancies between test versions must be carefully considered in cognitive profile analyses where index scores are directly contrasted, such as in the patterns of strengths and weaknesses used for the identification of specific learning disabilities (SLD), as described by Alfonso and Flanagan (2018). Clinicians operating under guidelines that mandate profile-based models must therefore confront these instrumentation artifacts during longitudinal evaluations. In summary, the present study suggests that while the AWM could be structurally identified as a distinct nested latent factor, its extreme collinearity with the superordinate WM factor implies that the AWM index score may only provide limited incremental utility for clinical and diagnostic decision-making beyond what is already captured by the WMI.

### 4.2 Structural validity of the Quantitative Reasoning index (QR)

As part of the first objective, results of the present CFAs failed to provide evidence for an independent dimension of the *Quantitative Reasoning* index (QR). Unlike a stable parameter estimation for model M2f _(AWM)_, expanding the primary measurement model of the German WISC-V to include an additional latent factor for QR resulted in the improper and unstable model M2f _(AWM, QR)_ and M2f* _(AWM, QR)_. Specifically, within the hierarchical model framework, the inclusion of the QR factor yielded a statistically inadmissible parameter estimation in the form of a severe localized Heywood case (i.e., negative residual variance). Within the bifactor model framework, model M2f* _(AWM, QR)_ completely failed to achieve mathematical convergence. Replicating the findings reported by McGill et al. (2025) for the US normative data, these critical estimation failures indicate that a distinct dimension for QR is structurally unviable within the German WISC-V.

Such mathematical estimation failures may serve as an informative psychometric finding, as they provide empirical evidence that the German WISC-V standardization sample cannot support the extraction of an independent QR factor, thereby exposing a severe over-factoring artifact. These structural limitations may be directly associated with the structural viability of *Fluid Reasoning* (FR) from which QR is conceptually presumed to branch. Since QR is defined as a narrow ability nested within the broader FR domain, identifying QR as a viable latent dimension inherently requires that a latent factor for FR itself can be robustly identified. However, this assumption has been repeatedly challenged in analyses on the WISC-V primary measurement model (e.g., Canivez et al., 2016, 2017). Similar findings for the German WISC-V (Pauls and Daseking, 2021; Pauls et al., 2020) further support that FR remains, at best, a highly fragile broad ability heavily saturated by *g*, thus not allowing for the extraction of an additional, subordinate QR factor. Historically, retaining a distinct FR factor on recent Wechsler scales has often necessitated complex statistical specifications that deviate from the test publisher approach, such as the correlated residuals between the latent factors FR and VS introduced by Reynolds and Keith (2017), instead of recombining both into a unified PR factor consistent with prior Wechsler editions. As a consequence, the FR factor cannot explain sufficient unique variance to be further partialed into a QR factor, thus likely representing an artificial latent dimension. While the AWM could at least be structurally identified as a distinct nested latent factor, the integration of a QR factor resulted in an inadmissible model solution, demonstrating that the associated index score fails to reflect a viable subdomain of FR. Although evaluating quantitative reasoning ability within educational settings may be intuitively appealing to clinicians, diagnostic decision-making based on the QR index scores of the German WISC-V alone is empirically unsupported by the findings of the present study and is therefore not recommended for clinical practice.

### 4.3 Structural configuration and dimensionality of the Nonverbal Index (NVI)

Regarding the second objective, the present CFAs provided robust empirical support for the hierarchical structure of the *Nonverbal Index* (NVI), successfully explaining the variance of the six nonverbal WISC-V subtest indicators through the first-order factors FR and VS that loaded on the superordinate second-order factor for nonverbal ability (NV). Due to its favorable global fit indices, model M3b suggests that the NVI scale provides clinicians with a psychometrically sound alternative to the FSIQ when estimating general cognitive ability. This structural alignment is further reflected in the strong correlation between the scaled scores for FSIQ and the NVI (*r* = 0.92) in the extended German standardization sample analyzed. Consequently, clinicians may interpret the NVI with confidence in specialized diagnostic situations where a nonverbal estimation of general intelligence is diagnostically indicated, such as the assessment of culturally and linguistically diverse populations, as well as individuals with speech or language impairments (Ortiz, 2023).

Although the NVI is structurally valid and represents an alternative measure to the FSIQ for nonverbal assessments, the multidimensionality of this ancillary index scale necessitates a cautious diagnostic approach. Given the substantial factor loadings of FR and VS on the superordinate NV factor, the NVI aggregates vast variance from both domains and might thus obscure meaningful score variations if a substantial discrepancy exists between fluid reasoning and visual–spatial capabilities. Crucially, however, the present model-based reliability analyses demonstrated that once the variance of the superordinate NV factor is controlled, the specific group factors for FR and VS failed to establish structural stability or retain sufficient unique, reliable variance. From a clinical perspective, these findings suggest that the nonverbal subtests in the WISC-V lack the statistical autonomy required to generate specific composite scores for FR and VS independent of global nonverbal ability. Consequently, attempting to isolate or interpret specific subscale profiles within this framework is empirically unsupported, as the specific index scale scores reflect global nonverbal ability rather than unique, distinct cognitive dimensions. Therefore, clinicians should focus their diagnostic interpretation strictly on the NVI as a unified and psychometrically robust marker of nonverbal cognitive ability.

### 4.4 Dimensionality of the General Ability Index (GAI) and Cognitive Proficiency Index (CPI)

The present CFAs provided structural support that the dimensionality of the *General Ability Index* (GAI) and the *Cognitive Proficiency Index* (CPI) is best represented by a hierarchical four-factor model solution. Encompassing nine subtest indicators, the corresponding model M4d specifies that the variance of eight of these subtest indicators is sufficiently explained by four first-order factors: VC and FR loading on a general ability factor (GA) as the second-order factor, alongside WM and PS loading on a cognitive proficiency factor (CP) as the second-order factor. Global fit indices for M4d confirm that establishing the GA and the CP as separate but intercorrelated superordinate latent factors indirectly accounts for the variance of their respective subtest indicators, validating both as psychometrically plausible index scales.

As pointed out by McGill et al. (2025), however, their structural configuration lacks an independent relationship with *g* (as per Zhu, 2009), thus making clinical inferences derived from discrepancies with the Full Scale IQ (FSIQ) highly speculative. This structural ambiguity is also supported by the present model-based reliability analyses, indicating that only the superordinate GA factor established robust structural stability and high construct replicability. In contrast, the CP dimension lacks the necessary homogeneity and construct replicability across samples, as its reliable variance is almost exclusively driven by processing speed while the working memory facet collapses almost entirely. Historically developed for pragmatic reasons rather than theoretical necessity, the GAI and CPI scales are frequently used to invalidate the FSIQ as a global measure based on significant GAI/CPI scatter—a heuristic that lacks empirical support (McGill, 2016) and is explicitly discouraged by Weiss et al. (2016). Technically, the FSIQ does not provide substantial diagnostic advantage over the GAI when estimating general intelligence (Farmer et al., 2020). Yet, because individual-level comparability between different global composite scores derived from the same test battery remains unsatisfactory (Grieder et al., 2022), clinicians should avoid uncritically selecting the highest available index scores or just those confirming their presumptions. In clinical practice, deviating from using the FSIQ in favor of alternative global ancillary index scales such as the GAI should always be carefully considered and be based on a compelling and pre-specified justification rooted in the specific strengths and limitations of the individual tested (Weiss et al., 2016).

### 4.5 Study limitations

Although findings of the present study are based on a large, population-representative German standardization sample, future research is strongly encouraged to further determine whether the WISC-V ancillary index scales are useful for diagnostic and clinical decision-making. As the validity of structural equation models is strictly constrained by the quality of the specific dataset analyzed, there are limitations to the information provided by CFA in isolation (Fried, 2020). While certain latent dimensions, such as quantitative reasoning (QR), failed to achieve stable parameter estimations or mathematical convergence in this extended German standardization sample, these constructs cannot be entirely dismissed as non-existent, as they might dynamically emerge within targeted clinical populations or alternative standardization samples (Dombrowski and McGill, 2024). Indeed, recent evidence demonstrates that construct validity and the stability of Wechsler factor configurations can vary substantially across different cognitive ability levels, with factor structures exhibiting increased fragility or non-convergence in low-IQ populations (Çelik et al., 2026). Because representative standardization samples naturally encompass a heterogeneous range of intellectual abilities, the inclusion of low-IQ variance within the normative population may have further contributed to the structural instability of the newly specified minor factor configurations like the QR, which branch from already fragile broad ability domains. Consequently, evaluating alternative configurations across distinct IQ strata remains an important caveat for future structural research. It should be noted, however, that the mathematical non-convergence found in the present study should be treated as an informative empirical benchmark rather than a methodological defect. When complex structural factor models collapse or fail to converge on highly powered, representative standardization samples, it signals an unvarnished psychometric boundary. Future research evaluating the WISC-V ancillary index scales should therefore transparently report these estimation limits, as they provide critical guardrails against publication bias and prevent the artificial forcing of model solutions that do not reflect genuine latent independence. Moreover, future research should address the necessity for cross-cultural replication utilizing different international adaptations of the WISC-V. Since minor variations in administration, language translation, and changes in item parameters can alter the covariance matrix of a complex test battery like the WISC-V, the structural validity of the ancillary index scales must be independently verified across different international versions before measurement invariance can be assumed.

In general, a broader methodological limitation stems from the exclusive reliance on the extended German standardization sample from a cross-sectional design standpoint. As critically highlighted in similar structural investigations on the German WISC-V (e.g., Pauls and Daseking, 2021; Pauls et al., 2020), standardization samples in general represent healthy populations that are fundamentally optimized for maximum variance and normal distributions. Consequently, the respective data contain an inherent upper limit regarding what can be uncovered about actual clinical utility. This also applies to the present model-based reliability analyses, which are strictly bounded by the non-clinical nature of the standardization sample under investigation. Future research should thus investigate whether the reliability coefficients of ancillary index scales remain equally fragile or dynamically alter when tested within targeted clinical populations. Additional evidence is therefore required for specific clinical populations to demonstrate whether ancillary index scales possess incremental validity and stability for clinical practice. In summary, future research should directly contrast the model solutions and reliability coefficients identified within selected clinical samples—such as children with specific learning disabilities—to test for measurement invariance and evaluate the construct validity of the ancillary index scales against objective external criteria (e.g., longitudinal performance outcomes or measures of school achievement).

## 5 Conclusion

To make valid and confident clinical judgments based on WISC-V index scores, clinicians must thoroughly understand the multidimensional architecture of the constructs they assess and avoid assuming that a score’s nominal label guarantees psychological unidimensionality without ambiguity (Haynes et al., 2019; McGill et al., 2018). The findings of the present study on the extended German standardization sample provide critical information regarding the structural validity and diagnostic viability of the German WISC-V ancillary index scales, yielding important convergences with the findings reported by McGill et al. (2025) on the US normative data. Consistent with McGill et al. (2025), the *Auditory Working Memory* index (AWM) was shown to be structurally identifiable but psychometrically redundant, thus offering almost no incremental utility beyond the *Working Memory Index* (WMI). Since a fluid reasoning factor (FR) has previously been found to lack sufficient unique variance, an additional subordinate dimension for quantitative reasoning (QR) could not be supported in the present study. Aligning closely with the findings of McGill et al. (2025), the *Nonverbal Index* (NVI), *General Ability Index* (GAI), and *Cognitive Proficiency Index* (CPI) are represented by plausible latent dimensions, yet their statistical proximity to the FSIQ and their risk of obscuring meaningful internal profile variation warrant a cautious approach.

These psychometric boundaries have distinct implications for local evidence-based practice. In contrast to the US diagnostic environment, European and German clinical guidelines (e.g., for neurodevelopmental and specific learning disorders) often place high interpretive weight on specialized cognitive profiles. The present analyses demonstrated that while the ancillary indices are methodologically transparent, clinicians must exercise caution when utilizing specific index scores in diagnostic decision-making. This is because their unique explanatory power and reliability may be heavily saturated by the overarching superordinate constructs, thus limiting their incremental utility beyond the global composites (e.g., FSIQ). In order for any ancillary index scales to be reliably interpreted in clinical practice according to established test standards (e.g., AERA, APA, and NCME, 2014), future research should focus strictly on empirical validation across diverse clinical populations. As long as further compelling evidence for the clinical validity of the ancillary index scales remains absent for the German WISC-V, both the interpretation of these scores and subsequent diagnostic decision-making should be approached with caution.

## Data Availability

The datasets presented in this article are not readily available because all rights for the raw data are reserved by the publisher of the WISC-V. Other data supporting the conclusions of this article will be made available by the authors with prior permission. Requests to access the datasets should be directed to paulsf@hsu-hh.de.
