# Supplementary material for: Structural validity of the German WISC-V beyond primary assessment: a factor-analytical approach to the ancillary index scales
Source: Front Psychol. 2026 Sep 3;17:1913590. doi: 10.3389/fpsyg.2026.1913590 (PMC13582241; doi:10.3389/fpsyg.2026.1913590)
Supplement: Supplementary file 1 [file Table_1.docx]

**Supplementary Tables**

| **Table S1.** | | | | | | | | | | | | |
| --- | --- | --- | --- | --- | --- | --- | --- | --- | --- | --- | --- | --- |
| *Specification and visual representation of the organization and scoring framework of the WISC-V.* | | | | | | | | | | | | |
| WISC-V subtest | Primary index / FSIQ | | | | | |  | Ancillary index | | | | |
|  | VCI | VSI | FRI | WMI | PSI | FSIQ |  | QR | AWM | NVI | GAI | CPI |
| Primary: |  |  |  |  |  |  |  |  |  |  |  |  |
| SI | ✓ |  |  |  |  | ✓ |  |  |  |  | ✓ |  |
| VO | ✓ |  |  |  |  | ✓ |  |  |  |  | ✓ |  |
| BD |  | ✓ |  |  |  | ✓ |  |  |  | ✓ | ✓ |  |
| VP |  | ✓ |  |  |  |  |  |  |  | ✓ |  |  |
| MR |  |  | ✓ |  |  | ✓ |  |  |  | ✓ | ✓ |  |
| FW |  |  | ✓ |  |  | ✓ |  | ✓ |  | ✓ | ✓ |  |
| DS |  |  |  | ✓ |  | ✓ |  |  | ✓ |  |  | ✓ |
| PS |  |  |  | ✓ |  |  |  |  |  | ✓ |  | ✓ |
| CD |  |  |  |  | ✓ | ✓ |  |  |  | ✓ |  | ✓ |
| SS |  |  |  |  | ✓ |  |  |  |  |  |  | ✓ |
| Secondary: |  |  |  |  |  |  |  |  |  |  |  |  |
| IN |  |  |  |  |  |  |  |  |  |  |  |  |
| CO |  |  |  |  |  |  |  |  |  |  |  |  |
| PC^a^ |  |  |  |  |  |  |  |  |  |  |  |  |
| AR |  |  |  |  |  |  |  | ✓ |  |  |  |  |
| LN |  |  |  |  |  |  |  |  | ✓ |  |  |  |
| CA |  |  |  |  |  |  |  |  |  |  |  |  |

*Note.* Primary subtests: SI = Similarities, VO = Vocabulary, BD = Block Design, VP = Visual Puzzles, MR = Matrix Reasoning, FW = Figure Weights, DS = Digit Span, PS = Picture Span, CD = Coding, SS = Symbol Search. Secondary subtests: IN = Information, CO = Comprehension, PC = Picture Concepts, AR = Arithmetic, LN = Letter-Number Sequencing, CA = Cancellation. Primary indices: VCI = Verbal Comprehension Index, VSI = Visual Spatial Index, FRI = Fluid Reasoning Index, WMI = Working Memory Index, PSI = Processing Speed Index, FSIQ = Full Scale IQ. Ancillary indices: QR = Quantitative Reasoning, AWM = Auditory Working Memory, NVI = Nonverbal Index, GAI = General Ability Index, CPI = Cognitive Proficiency Index. ^a^ The subtest Picture Concepts (PC) was excluded from the German WISC-V according to a standardization kit that provides the basic framework for all European WISC-V versions.

provides the basic framework for all European WISC-V versions

| **Table S2.** | | | | | | | | | | | | | | | | | | | | | | | | |
| --- | --- | --- | --- | --- | --- | --- | --- | --- | --- | --- | --- | --- | --- | --- | --- | --- | --- | --- | --- | --- | --- | --- | --- | --- |
| *Specification and visual representation of the structural framework of competing hierarchical four-factor CFA models based on the German WISC-V primary and secondary subtests.* | | | | | | | | | | | | | | | | | | | | | | | | |
| WISC-V subtest |  | | | |  | Hierarchical four-factor model | | | | | | | | | | | | | | | | | | |
|  | Baseline 4-factor | | | |  | M1a | | | |  | M1b | | | |  | M1c | | | |  | M1d | | | |
|  | F1 | F2 | F3 | F4 |  | F1 | F2 | F3 | F4 |  | F1 | F2 | F3 | F4 |  | F1 | F2 | F3 | F4 |  | F1 | F2 | F3 | F4 |
| SI | • |  |  |  |  | • |  |  |  |  | • |  |  |  |  | • |  |  |  |  | • |  |  |  |
| VO | • |  |  |  |  | • |  |  |  |  | • |  |  |  |  | • |  |  |  |  | • |  |  |  |
| IN | • |  |  |  |  | • |  |  |  |  | • |  |  |  |  | • |  |  |  |  | • |  |  |  |
| CO | • |  |  |  |  | • |  |  |  |  | • |  |  |  |  | • |  |  |  |  | • |  |  |  |
| BD |  | • |  |  |  |  | • |  |  |  |  | • |  |  |  |  | • |  |  |  |  | • |  |  |
| VP |  | • |  |  |  |  | • |  |  |  |  | • |  |  |  |  | • |  |  |  |  | • |  |  |
| MR |  | • |  |  |  |  | • |  |  |  |  |  | • |  |  |  | • |  |  |  |  | • |  |  |
| FW |  | • |  |  |  |  | • |  |  |  |  |  | • |  |  |  | • |  |  |  |  | • |  |  |
| AR |  |  | • |  |  |  |  | • |  |  |  |  | • |  |  |  | • | • |  |  | • | • | • |  |
| DS |  |  | • |  |  |  |  | • |  |  |  |  | • |  |  |  |  | • |  |  |  |  | • |  |
| PS |  |  | • |  |  |  |  | • |  |  |  |  | • |  |  |  |  | • |  |  |  |  | • |  |
| LN |  |  | • |  |  |  |  | • |  |  |  |  | • |  |  |  |  | • |  |  |  |  | • |  |
| CD |  |  |  | • |  |  |  |  | • |  |  |  |  | • |  |  |  |  | • |  |  |  |  | • |
| SS |  |  |  | • |  |  |  |  | • |  |  |  |  | • |  |  |  |  | • |  |  |  |  | • |
| CA |  |  |  | • |  |  |  |  | • |  |  |  |  | • |  |  |  |  | • |  |  |  |  | • |
| *g* |  |  |  |  |  | • | • | • | • |  | • | • | • | • |  | • | • | • | • |  | • | • | • | • |

*Note.* Baseline 4-factor = first-order oblique four-factor model with intercorrelated first-order factors. M1a to M1d are specified according to the hierarchical models reported in the German WISC-V *Technical and Interpretive Manual* and represent hierarchical models based on 15 primary and secondary subtests loading on four first-order factors, which load on one second-order factor. Subtests: SI = Similarities, VO = Vocabulary, IN = Information, CO = Comprehension, BD = Block Design, VP = Visual Puzzles, MR = Matrix Reasoning, FW = Figure Weights, AR = Arithmetic, DS = Digit Span, PS = Picture Span, LN = Letter-Number Sequencing, CD = Coding, SS = Symbol Search, CA = Cancellation. First-order factors: F1 = Verbal Comprehension (VC), F2 = Perceptual Reasoning (PR), F3 = Working Memory (WM), F4 = Processing Speed (PS). Second-order factor: *g* = General intelligence.

| **Table S3.** | | | | | | | | | | | | | | | | | | | | | | | |
| --- | --- | --- | --- | --- | --- | --- | --- | --- | --- | --- | --- | --- | --- | --- | --- | --- | --- | --- | --- | --- | --- | --- | --- |
| *Specification and visual representation of the structural framework of competing bifactor four-factor CFA models based on the German WISC-V primary and secondary subtests.* | | | | | | | | | | | | | | | | | | | | | | | |
| WISC-V subtest | Bifactor four-factor model | | | | | | | | | | | | | | | | | | | | | | |
|  | M1a* | | | | |  | M1b* | | | | |  | M1c* | | | | |  | M1d* | | | | |
|  | F1 | F2 | F3 | F4 | *g* |  | F1 | F2 | F3 | F4 | *g* |  | F1 | F2 | F3 | F4 | *g* |  | F1 | F2 | F3 | F4 | *g* |
| SI | • |  |  |  | • |  | • |  |  |  | • |  | • |  |  |  | • |  | • |  |  |  | • |
| VO | • |  |  |  | • |  | • |  |  |  | • |  | • |  |  |  | • |  | • |  |  |  | • |
| IN | • |  |  |  | • |  | • |  |  |  | • |  | • |  |  |  | • |  | • |  |  |  | • |
| CO | • |  |  |  | • |  | • |  |  |  | • |  | • |  |  |  | • |  | • |  |  |  | • |
| BD |  | • |  |  | • |  |  | • |  |  | • |  |  | • |  |  | • |  |  | • |  |  | • |
| VP |  | • |  |  | • |  |  | • |  |  | • |  |  | • |  |  | • |  |  | • |  |  | • |
| MR |  | • |  |  | • |  |  |  | • |  | • |  |  | • |  |  | • |  |  | • |  |  | • |
| FW |  | • |  |  | • |  |  |  | • |  | • |  |  | • |  |  | • |  |  | • |  |  | • |
| AR |  |  | • |  | • |  |  |  | • |  | • |  |  | • | • |  | • |  | • | • | • |  | • |
| DS |  |  | • |  | • |  |  |  | • |  | • |  |  |  | • |  | • |  |  |  | • |  | • |
| PS |  |  | • |  | • |  |  |  | • |  | • |  |  |  | • |  | • |  |  |  | • |  | • |
| LN |  |  | • |  | • |  |  |  | • |  | • |  |  |  | • |  | • |  |  |  | • |  | • |
| CD |  |  |  | • | • |  |  |  |  | • | • |  |  |  |  | • | • |  |  |  |  | • | • |
| SS |  |  |  | • | • |  |  |  |  | • | • |  |  |  |  | • | • |  |  |  |  | • | • |
| CA |  |  |  | • | • |  |  |  |  | • | • |  |  |  |  | • | • |  |  |  |  | • | • |

*Note.* M1a* to M1d* are specified according to the hierarchical models reported in the German WISC-V *Technical and Interpretive Manual* and represent bifactor models based on 15 primary and secondary subtests loading on four group factors and on one general factor. Subtests: SI = Similarities, VO = Vocabulary, IN = Information, CO = Comprehension, BD = Block Design, VP = Visual Puzzles, MR = Matrix Reasoning, FW = Figure Weights, AR = Arithmetic, DS = Digit Span, PS = Picture Span, LN = Letter-Number Sequencing, CD = Coding, SS = Symbol Search, CA = Cancellation. Group factors: F1 = Verbal Comprehension (VC), F2 = Perceptual Reasoning (PR), F3 = Working Memory (WM), F4 = Processing Speed (PS). General factor: *g* = General intelligence.

| **Table S4.** | | | | | | | | | | | | | | | | | | | | | | | | | | | | | | | | | | | | | | | | | | |  |
| --- | --- | --- | --- | --- | --- | --- | --- | --- | --- | --- | --- | --- | --- | --- | --- | --- | --- | --- | --- | --- | --- | --- | --- | --- | --- | --- | --- | --- | --- | --- | --- | --- | --- | --- | --- | --- | --- | --- | --- | --- | --- | --- | --- |
| *Specification and visual representation of the structural framework of competing hierarchical five-factor CFA models based on the German WISC-V primary and secondary subtests.* | | | | | | | | | | | | | | | | | | | | | | | | | | | | | | | | | | | | | | | | | | |  |
| WISC-V subtest |  | | | | |  | Hierarchical five-factor model | | | | | | | | | | | | | | | | | | | | | | | | | | | | | | | | | | | | |
|  | Baseline 5-factor | | | | |  | M2a | | | | |  | M2b | | | | |  | M2c | | | | |  | M2d | | | | |  | M2e | | | | |  | M2f ^a^ | | | | | |  |
|  | F1 | F2 | F3 | F4 | F5 |  | F1 | F2 | F3 | F4 | F5 |  | F1 | F2 | F3 | F4 | F5 |  | F1 | F2 | F3 | F4 | F5 |  | F1 | F2 | F3 | F4 | F5 |  | F1 | F2 | F3 | F4 | F5 |  | F1 | F2 | F3 | F4 | F5 | *g* |  |
| SI | • |  |  |  |  |  | • |  |  |  |  |  | • |  |  |  |  |  | • |  |  |  |  |  | • |  |  |  |  |  | • |  |  |  |  |  | • |  |  |  |  |  |  |
| VO | • |  |  |  |  |  | • |  |  |  |  |  | • |  |  |  |  |  | • |  |  |  |  |  | • |  |  |  |  |  | • |  |  |  |  |  | • |  |  |  |  |  |  |
| IN | • |  |  |  |  |  | • |  |  |  |  |  | • |  |  |  |  |  | • |  |  |  |  |  | • |  |  |  |  |  | • |  |  |  |  |  | • |  |  |  |  |  |  |
| CO | • |  |  |  |  |  | • |  |  |  |  |  | • |  |  |  |  |  | • |  |  |  |  |  | • |  |  |  |  |  | • |  |  |  |  |  | • |  |  |  |  |  |  |
| BD |  | • |  |  |  |  |  | • |  |  |  |  |  | • |  |  |  |  |  | • |  |  |  |  |  | • |  |  |  |  |  | • |  |  |  |  |  | • |  |  |  |  |  |
| VP |  | • |  |  |  |  |  | • |  |  |  |  |  | • |  |  |  |  |  | • |  |  |  |  |  | • |  |  |  |  |  | • |  |  |  |  |  | • |  |  |  |  |  |
| MR |  |  | • |  |  |  |  |  | • |  |  |  |  |  | • |  |  |  |  |  | • |  |  |  |  |  | • |  |  |  |  |  | • |  |  |  |  |  | • |  |  |  |  |
| FW |  |  | • |  |  |  |  |  | • |  |  |  |  |  | • |  |  |  |  |  | • |  |  |  |  |  | • |  |  |  |  |  | • |  |  |  |  |  | • |  |  |  |  |
| AR |  |  |  | • |  |  |  |  |  | • |  |  |  |  | • |  |  |  |  |  | • | • |  |  | • |  |  | • |  |  | • |  | • | • |  |  | • |  |  | • |  | • |  |
| DS |  |  |  | • |  |  |  |  |  | • |  |  |  |  |  | • |  |  |  |  |  | • |  |  |  |  |  | • |  |  |  |  |  | • |  |  |  |  |  | • |  |  |  |
| PS |  |  |  | • |  |  |  |  |  | • |  |  |  |  |  | • |  |  |  |  |  | • |  |  |  |  |  | • |  |  |  |  |  | • |  |  |  |  |  | • |  |  |  |
| LN |  |  |  | • |  |  |  |  |  | • |  |  |  |  |  | • |  |  |  |  |  | • |  |  |  |  |  | • |  |  |  |  |  | • |  |  |  |  |  | • |  |  |  |
| CD |  |  |  |  | • |  |  |  |  |  | • |  |  |  |  |  | • |  |  |  |  |  | • |  |  |  |  |  | • |  |  |  |  |  | • |  |  |  |  |  | • |  |  |
| SS |  |  |  |  | • |  |  |  |  |  | • |  |  |  |  |  | • |  |  |  |  |  | • |  |  |  |  |  | • |  |  |  |  |  | • |  |  |  |  |  | • |  |  |
| CA |  |  |  |  | • |  |  |  |  |  | • |  |  |  |  |  | • |  |  |  |  |  | • |  |  |  |  |  | • |  |  |  |  |  | • |  |  |  |  |  | • |  |  |
| *g* |  |  |  |  |  |  | • | • | • | • | • |  | • | • | • | • | • |  | • | • | • | • | • |  | • | • | • | • | • |  | • | • | • | • | • |  | • | • | • | • | • |  |  |

*Note*. Baseline 5-factor = first-order oblique five-factor model with intercorrelated first-order factors. M2a to M2e are specified according to the hierarchical models reported in the German WISC-V *Technical and Interpretive Manual* and represent hierarchical models based on 15 primary and secondary subtests loading on five first-order factors, which load on one second-order factor. Subtests: SI = Similarities, VO = Vocabulary, IN = Information, CO = Comprehension, BD = Block Design, VP = Visual Puzzles, MR = Matrix Reasoning, FW = Figure Weights, AR = Arithmetic, DS = Digit Span, PS = Picture Span, LN = Letter-Number Sequencing, CD = Coding, SS = Symbol Search, CA = Cancellation. First-order factors: F1 = Verbal Comprehension (VC), F2 = Visual Spatial (VS), F3 = Fluid Reasoning (FR), F4 = Working Memory (WM), F5 = Processing Speed (PS). Second-order factor: *g* = General intelligence. ^a^ Hierarchical five-factor model specified according to Reynolds and Keith (2017) with an additional correlation between FR and VS and a direct loading of AR on *g.*

| **Table S5.** | | | | | | | | | | | | | | | | | | | | | | | | | | | | | | | | | | | | | | | | | |
| --- | --- | --- | --- | --- | --- | --- | --- | --- | --- | --- | --- | --- | --- | --- | --- | --- | --- | --- | --- | --- | --- | --- | --- | --- | --- | --- | --- | --- | --- | --- | --- | --- | --- | --- | --- | --- | --- | --- | --- | --- | --- |
| *Specification and visual representation of the structural framework of competing bifactor five-factor CFA models based on the German WISC-V primary and secondary subtests.* | | | | | | | | | | | | | | | | | | | | | | | | | | | | | | | | | | | | | | | | | |
| WISC-V subtest | Bifactor five-factor model | | | | | | | | | | | | | | | | | | | | | | | | | | | | | | | | | | | | | | | | |
|  | M2a* | | | | | |  | M2b* | | | | | |  | M2c* | | | | | |  | M2d* | | | | | |  | M2e* | | | | | |  | M2f* ^a^ | | | | | |
|  | F1 | F2 | F3 | F4 | F5 | *g* |  | F1 | F2 | F3 | F4 | F5 | *g* |  | F1 | F2 | F3 | F4 | F5 | *g* |  | F1 | F2 | F3 | F4 | F5 | *g* |  | F1 | F2 | F3 | F4 | F5 | *g* |  | F1 | F2 | F3 | F4 | F5 | *g* |
| SI | • |  |  |  |  | • |  | • |  |  |  |  | • |  | • |  |  |  |  | • |  | • |  |  |  |  | • |  | • |  |  |  |  | • |  | • |  |  |  |  | • |
| VO | • |  |  |  |  | • |  | • |  |  |  |  | • |  | • |  |  |  |  | • |  | • |  |  |  |  | • |  | • |  |  |  |  | • |  | • |  |  |  |  | • |
| IN | • |  |  |  |  | • |  | • |  |  |  |  | • |  | • |  |  |  |  | • |  | • |  |  |  |  | • |  | • |  |  |  |  | • |  | • |  |  |  |  | • |
| CO | • |  |  |  |  | • |  | • |  |  |  |  | • |  | • |  |  |  |  | • |  | • |  |  |  |  | • |  | • |  |  |  |  | • |  | • |  |  |  |  | • |
| BD |  | • |  |  |  | • |  |  | • |  |  |  | • |  |  | • |  |  |  | • |  |  | • |  |  |  | • |  |  | • |  |  |  | • |  |  | • |  |  |  | • |
| VP |  | • |  |  |  | • |  |  | • |  |  |  | • |  |  | • |  |  |  | • |  |  | • |  |  |  | • |  |  | • |  |  |  | • |  |  | • |  |  |  | • |
| MR |  |  | • |  |  | • |  |  |  | • |  |  | • |  |  |  | • |  |  | • |  |  |  | • |  |  | • |  |  |  | • |  |  | • |  |  |  | • |  |  | • |
| FW |  |  | • |  |  | • |  |  |  | • |  |  | • |  |  |  | • |  |  | • |  |  |  | • |  |  | • |  |  |  | • |  |  | • |  |  |  | • |  |  | • |
| AR |  |  |  | • |  | • |  |  |  | • |  |  | • |  |  |  | • | • |  | • |  | • |  |  | • |  | • |  | • |  | • | • |  | • |  | • |  |  | • |  | • |
| DS |  |  |  | • |  | • |  |  |  |  | • |  | • |  |  |  |  | • |  | • |  |  |  |  | • |  | • |  |  |  |  | • |  | • |  |  |  |  | • |  | • |
| PS |  |  |  | • |  | • |  |  |  |  | • |  | • |  |  |  |  | • |  | • |  |  |  |  | • |  | • |  |  |  |  | • |  | • |  |  |  |  | • |  | • |
| LN |  |  |  | • |  | • |  |  |  |  | • |  | • |  |  |  |  | • |  | • |  |  |  |  | • |  | • |  |  |  |  | • |  | • |  |  |  |  | • |  | • |
| CD |  |  |  |  | • | • |  |  |  |  |  | • | • |  |  |  |  |  | • | • |  |  |  |  |  | • | • |  |  |  |  |  | • | • |  |  |  |  |  | • | • |
| SS |  |  |  |  | • | • |  |  |  |  |  | • | • |  |  |  |  |  | • | • |  |  |  |  |  | • | • |  |  |  |  |  | • | • |  |  |  |  |  | • | • |
| CA |  |  |  |  | • | • |  |  |  |  |  | • | • |  |  |  |  |  | • | • |  |  |  |  |  | • | • |  |  |  |  |  | • | • |  |  |  |  |  | • | • |

*Note.* M2a* to M2e* are specified according to the hierarchical models reported in the German WISC-V *Technical and Interpretive Manual* and represent bifactor models based on 15 primary and secondary subtests loading on five group factors and on one general factor. Subtests: SI = Similarities, VO = Vocabulary, IN = Information, CO = Comprehension, BD = Block Design, VP = Visual Puzzles, MR = Matrix Reasoning, FW = Figure Weights, AR = Arithmetic, DS = Digit Span, PS = Picture Span, LN = Letter-Number Sequencing, CD = Coding, SS = Symbol Search, and CA = Cancellation. Group factors: F1 = Verbal Comprehension (VC), F2 = Visual Spatial (VS), F3 = Fluid Reasoning (FR), F4 = Working Memory (WM), and F5 = Processing Speed (PS). General factor: *g* = General intelligence. ^a^ Bifactor five-factor model specified according to the hierarchical five-factor model by Reynolds and Keith (2017) with an additional correlation between FR and VS and a direct loading of AR on *g.*
